# FISH analysis of numerical chromosomal abnormalities in the sperm of robertsonian translocation der(13; 14)(q10;q10) carriers

**DOI:** 10.3389/fgene.2022.1010568

**Published:** 2022-09-27

**Authors:** Saijuan Zhu, Yong Zhu, Feng Zhang, Junping Wu, Ying Chen, Yijuan Sun, Jing Fu, Jiangnan Wu, Min Xiao, Shuo Zhang, Jing Zhou, Caixia Lei, Feng Jiang

**Affiliations:** ^1^ Laboratory of Genetics, Shanghai Ji Ai Genetics and IVF Institute, Shanghai, China; ^2^ Human Sperm Bank, Fudan University, Shanghai, China; ^3^ Laboratory of Andrology, Shanghai Ji Ai Genetics and IVF Institute, Shanghai, China; ^4^ Laboratory of Embryology, Shanghai Ji Ai Genetics and IVF Institute, Shanghai, China; ^5^ Department of Clinical Epidemiology, Clinical Research Unit, Obstetrics and Gynecology Hospital, Fudan University, Shanghai, China

**Keywords:** inter-chromosomal effect, robertsonian translocation, spermatozoa, meiotic segregation, aneuploidy

## Abstract

Fluorescence *in situ* hybridization analysis of numerical chromosomal abnormalities in the sperm of Robertsonian translocation der (13;14) (q10;q10) carriers has focused on a limited number of chromosomes mainly on chromosome 13, 18, 21, X, and Y. Here, we aimed to expand the analysis to all chromosomes by increasing the number of probes analyzed in fluorescence *in situ* hybridization. The incidence of numerical abnormalities of all chromosomes (1–22, X, and Y) was determined in sperm from 10 carriers of the Robertsonian translocation der(13;14)(q10;q10) and 10 normozoospermic males to fully assess the effect of translocation-derived chromosome on the segregation of all chromosomes during meiosis. Numerical abnormalities of the two translocated chromosomes were frequently detected in the sperm of der (13;14) translocation carriers, with an average frequency of 14.55% ± 6.00% for chromosome 13 and 13.27% ± 4.14% for chromosome 14. Numerical abnormalities of nontranslocated chromosomes, with an average frequency of 1.77% ± 0.62% (range, 1.16%–3.73%), was lower than that of translocated chromosome. However, the cumulative numerical abnormality of the 22 nontranslocated chromosomes was comparable to that of the two translocated chromosomes. Significantly increased numerical abnormalities in der(13;14) translocation carriers compared with those in normozoospermic males indicates the presence of translocation-derived chromosome disturbances, with translocated chromosomes being most affected; nontranslocated chromosomes were also affected, but to a lesser extent due to a mild interchromosomal effect.

## Introduction

A Robertsonian translocation is a chromosomal abnormality in which two proximal acrocentric chromosomes break at or near the centromere and fuse at the break site. The incidence of Robertsonian translocations in newborns is 1.23/1,000 (Poot and Hochstenbach, 2021). Humans have 5 proximal acrocentric chromosomes (13, 14, 15, 21, and 22) that can fuse to form 10 heterologous and 5 homologous Robertsonian translocation pairs. The most common Robertsonian translocation occurs between chromosomes 13 and 14, accounting for 75% of all Robertsonian translocations (Poot and Hochstenbach, 2021). In der(13;14) translocation, one chromosome 13 and one chromosome 14 lose their short arm during translocation, and the remaining two long arms fuse together to form a derivative chromosome. Because these chromosomes fuse into a single chromosome, the nuclei of Robertsonian translocation carriers have 45 instead of 46 chromosomes. However, the two long arms that form the derivative chromosome contain nearly all the genes of the two proximal acrocentric chromosomes. Therefore, Robertsonian translocation carriers often do not have a clinical phenotype, despite the reduced number of chromosomes in the nucleus (Godo et al., 2015). However, spermatocytes of male carriers may produce gametes with an abnormal number of chromosomes.

During meiosis in spermatogenesis, the derivative chromosome, formed by the long arms of chromosomes 13 and 14, pairs up with the two normal chromosomes 13 and 14, forming an asymmetric structure called a trivalent. The trivalent structure interferes with the segregation of chromosomes 13 and 14, making them segregate through modes of alternate, adjacent I, adjacent II, and 3:0, which produce gametes with different numbers of chromosomes 13 and 14 (Godo et al., 2015). Alternate segregation produces balanced gametes with two chromosomes 13 and 14. Conversely, the other three segregation modes produce unbalanced gametes. Adjacent segregation produces four aneuploid gametes (nullisomy 13, disomy 13, nullisomy 14, and disomy 14), while 3:0 segregation produces two types of aneuploid gametes (double nullisomy 13 and 14, and double disomy 13 and 14).

The gamete frequencies of the different segregation modes have been extensively analyzed (Escudero et al., 2000; Frydman et al., 2001; Anton et al., 2004; Brugnon et al., 2006; Ogur et al., 2006; Kekesi et al., 2007; Cassuto et al., 2011; Ferfouri et al., 2011; Mahjoub et al., 2011; Perrin et al., 2013; Pylyp et al., 2013; Rouen et al., 2013; Vozdova et al., 2013; Godo et al., 2015; Wang et al., 2017; Lamotte et al., 2018). The segregation modes have been well-studied, with an average frequency of 79.3% ± 10.2%, 19.7% ± 10.2%, 0.8% ± 1.0%, and 0.2 ± 0.2% for alternate, adjacent, 3:0, and “others,” respectively (Wiland et al., 2020).However, most studies used a dual-color probe set, containing two probes specific for chromosomes 13 and 14, and were unable to distinguish 3:0-separated sperm from diploid sperm or sperm that had failed to hybridize, meaning that a portion of the 3:0 sperm was diploid or failed-to-hybridize sperms. Conversely, the tri-color probe set, which was built by adding an autosomal probe, as an internal ploidy control signal, to a dual-color probe set, can distinguish diploid and unhybridized sperm from 3:0-separated sperm. As most studies have focused on the frequency of the different segregation modes, a few studies have reported on the frequency of aneuploidy, corresponding to the four specific segregations (Escudero et al., 2000; Anton et al., 2004; Mahjoub et al., 2011; Pylyp et al., 2013; Lamotte et al., 2018). By pooling their results, The average frequency of numerical abnormalities was 22.40% ± 8.49% and the average frequency of disomy 13, nullisomy 13, disomy 14, and nullisomy 14 was 4.82% ± 2.65%, 5.26% ± 2.69%, 4.45% ± 2.47%, and 5.40% ± 2.67%, respectively (Supplementary Table S1). Because this needs to be verified, in this study, we analyzed the frequency of aneuploidy for chromosomes 13 and 14, using a tri-color probe set, in which an additional autosomal probe was added.

The analysis of numerical abnormalities for nontranslocated chromosomes has not been as extensive as that for chromosome 13 and 14. Mainly focusing on chromosomes 18, 21, 22, X, and Y (other chromosomes are rarely involved), researchers explored the possible interchromosomal effect (ICE) in male carriers of der (13;14) translocation (Hajlaoui et al., 2018; Wiland et al., 2020). The ICE was first proposed by Lejeune in 1963 (Lejeune, 1963). Lejeune found that a mother with a balanced chromosomal translocation gave birth to a child with Turner syndrome and speculated that the mother’s translocation may lead to other chromosomal aneuploidies, not just aneuploidies of the two translocated chromosomes, in children. In other words, the chromosome derived from translocation fusion interferes with the segregation of other nontranslocated chromosomes, increasing the risk of other chromosomes mis-segregating during meiosis, a phenomenon known as the ICE (Lejeune, 1963; Balasar and Acar, 2020; Miller, 2020).

Previous studies have investigated the ICE by performing fluorescence *in situ* hybridization (FISH) in sperm. Some studies showed the presence of an ICE (Cassuto et al., 2011; Olszewska et al., 2021), whereas others did not (Pylyp et al., 2013; Lamotte et al., 2018). Most studies found an ICE on some chromosomes in some carriers, but not in others (Therman et al., 1989; Morel et al., 2001; Baccetti et al., 2005; Machev et al., 2005; Chen et al., 2007; Perrin et al., 2013; Balasar and Acar, 2020; Olszewska et al., 2021), strongly suggesting carrier- and chromosome-specific characteristics of the ICE. However, the chromosome-specific characteristics are not fully understood, and it is not yet clear which nontranslocated chromosomes are prone to the ICE. This may be because not all nontranslocated chromosomes are analyzed for the presence of the ICE. The analysis of all nontranslocated chromosomes requires multiple rounds of experiments because of the limited number of fluorescent molecules and space within sperm nuclei, which can cause fluorescence signals to overlap, complicating the analysis. Given this, the cost of the probe, and the analytical workload to detect all non-translocated chromosomes, in most studies, only a few specific chromosomes were selected for analysis. Despite the difficulty in analyzing all nontranslocated chromosomes, the results are valuable for a comprehensive study of the ICE in Robertsonian translocations.

Therefore, we aimed to assess the numerical abnormalities of the translocated chromosomes, the presence of the ICE, and its chromosome-specific characteristics in the sperm of der (13;14) translocation carriers by performing aneuploidy analysis of all chromosomes (1–22, X, and Y).

## 2 Materials and methods

### 2.1 Subjects

Ten der (13;14) translocation carriers (age, 33.0 ± 2.9 [range, 28–38] years) were recruited from Shanghai Ji Ai Genetics & IVF Institute, Obstetrics & Gynecology Hospital of Fudan University. According to the fifth edition of the World Health Organization Laboratory Manual for the Examination and Processing of Human Semen (2010) (Cooper et al., 2010), six carriers had teratozoospermia, three had oligoasthenoteratozoospermia, and one had severe oligozoospermia. The severe oligozoospermic patient had very low sperm counts, with one to two sperm observed per high power field (1-2 sperm/HP), ever then, a sufficient number of sperm (approximate 22,613 sperm) were collected in the whole the semen for FISH experiments.

Ten normozoospermic donors [age, 30.7 ± 4.7 (range, 25–39) years] in the Human Sperm Bank of Fudan University, who met the sperm donation criteria, were included as controls. All donors presented normal karyotype without der(13;14) translocation or any other structural abnormalities. All donors had normal semen parameters, including sperm concentration, progressive motility, and morphology. The semen parameters of the 10 der(13;14) translocation carriers and 10 control donors are listed in Supplementary Table S2.

### 2.2 Ethical approval

All participants underwent genetic counseling and provided informed consent regarding donation and the purpose of the sperm aneuploidy study program. The study was approved by the Ethics Committees of Shanghai JiAi Genetics and IVF Institute (JIAI E2018-23) and the Ethics Committees of the Human Sperm Bank of Fudan University (HSBOFU 2021-01).

### 2.3 Semen preparation

Semen was collected after 3 days of abstinence, washed thrice in 1 × Dulbecco’s phosphate-buffered saline (Life Technologies, Carlsbad, CA, United States), and centrifuged at 300 ×*g* for 8 min. Sperm cells were transferred to glass slides and digested with 1N NaOH for 2 min to decondense the highly compacted sperm chromatin. The slides were dehydrated with 70%, 85%, and 100% ethanol for 1 min each and air-dried.

### 2.4 Fluorescence *in situ* hybridization protocol

Probe mix solutions were prepared according to the manufacturer’s instructions (Abbott Molecular, Inc.). For each carrier, a single round of FISH was performed on translocated chromosomes, with approximately 1,000 sperm nuclei scored per chromosome using a tri-color probe mix. The tri-color probe set consisted of an RBI locus-specific probe for chromosome 13, with green fluorescence, a subtelomeric-specific probe for chromosome 14, with orange fluorescence, and a centromere-specific probe for chromosome 18, with aquar fluorescence, serving as the internal ploidy control. In addition, 20 rounds of FISH experiment were performed for nontranslocated chromosomes on 20 separate slides, and each nontranslocated chromosome was analyzed by scoring approximately 1,000 sperm cells from the corresponding slide hybridized with a specific dual- or tri-color probe mix. The tri-color probe mix for chromosomes 1–4, 6–12, and 16–18 contained centromeric probes for the corresponding chromosomes (1–4, 6–12, 16–17 or 18) and centromeric probes for the chromosomes X and Y serving as diploidy control. The dual-color probe mix for chromosomes 5, 15, 19, 20, and 22 contained subtelomeric probes for the corresponding chromosomes (5, 15, 19, 20, or 22) and a centromeric probe for chromosome 18 serving as ploidy control. The dual-color mix for chromosome 21 contained D21S341, D21S342 locus-specific probe for chromosome 21 and a centromeric probe for chromosome 18 served as a ploidy control. The X and Y chromosome were analyzed using a tri-color probe set, which consisted of the sex chromosomes and an autosomal probe serving as the ploidy control. The probe sets are listed in Supplementary Table S3.

For each donor, 22 rounds of FISH were performed separately on 22 slides, with corresponding dual- or tri-color probe set, to assess all chromosomes; approximately 1,000 sperm nuclei were scored for each chromosome. The tri-color probe mix for chromosomes 1–4, 6–12, and 16–18 contained centromeric probes for the corresponding chromosomes (1–4, 6–12, 16–17, or 18) and centromeric probes for the X and Y chromosomes serving as diploidy control. The dual-color probe mix for chromosomes 5, 14–15, 19–20, and 22 contained subtelomeric probes for the corresponding chromosomes (5, 14–15, 19–20, or 22) and a centromeric probe for chromosome 18 serving as diploidy control. The dual-color mix for chromosome 13 contained an RBI locus-specific probe for chromosome 13 and a centromeric probe for chromosome 18 served as a ploidy control. The X and Y chromosome were analyzed using a tri-color probe set, which consisted of the sex chromosomes and an autosomal probe serving as the ploidy control. The probe sets are listed in Supplementary Table S3.

After mixing, the probe solution was added to the slides. The slides were covered with coverslips and sealed with rubber cement. Denaturation and hybridization were carried out at 78°C for 5 min and at 37°C overnight, respectively, in a ThermoBrite humidification chamber (IRIS International Inc., Chatsworth, CA, United States). After overnight hybridization, the slides were washed with 0.4 × SSC/0.3% IGEPAL at 72°C for 2 min, and with 2 × SSC/0.1% IGEPAL at room temperature for 1 min. The slides were air-dried, after which a 4, 6-diamine-2-phenylindole solution (Abbott Molecular, Inc.) was added. The samples were analyzed using an automated CytoVision image analysis and capture system (Leica Biosystems Richmond Inc., Richmond, IL, United States).

### 2.5 Scoring criteria

An intact nucleus, clear borders, and strong fluorescence signals were the criteria for sperm nuclei scoring. The definitions of various numerical abnormalities were as follows: 1) nullisomy, no signal for the given probe and one signal for the other control probe; 2) disomy, two signals for the given probe and one signal for the other control probe; 3) diploidy, two signals for both the given probe and the other control probe; and 4) “others” consisting of trisomy (three signals for the given probe and one signal for the other control probe), triploidy (three signals for both the given probe and the other control probe), tetraploidy (four signals for both the given probe and the other control probe), and multiple aneuploidies (in which the signals for the given probe and the other control probe differ from those described above). The signal patterns for the various numerical chromosomal abnormalities are shown in Supplementary Figure S1.

The scoring system was developed using an automated system (GSL-120; Leica Biosystems Richmond Inc.), with provision for automated slide loading, cell finding, image capturing, signal scanning, and sorting. The system captured approximately 1,000 sperm cells and sorted the different signal patterns. An experienced technician double-checked the signal from each cell, using the scoring criteria described above, to ensure classification accuracy.

### 2.6 Statistical analysis

The frequency of aneuploidy was calculated as the sum of the frequencies of nullisomy and disomy. The frequency of diploidy and “others” per carrier with nontranslocated chromosomes was calculated by dividing the number of diploid or “other” sperm cells by the total number of sperm counted for all 22 nontranslocated chromosomes, which excluded the two translocated chromosomes. The frequency of total numerical chromosomal abnormalities was calculated as the sum of the frequencies of aneuploidy, diploidy, and “others.” The frequency of diploidy and “others” per carrier with all chromosomes was calculated by dividing the number of diploid or “other” sperm cells by the total number of sperm counted for all chromosomes, which included the two translocated chromosomes. The overall frequency of nullisomy, disomy, and aneuploidy per carrier with nontranslocated chromosomes was calculated as the sum of the frequencies of the corresponding abnormalities of all 22 nontranslocated chromosomes, which excluded the two translocated chromosomes. The overall frequency of nullisomy, disomy, and aneuploidy per carrier with all chromosomes was calculated as the sum of the frequencies of the corresponding abnormalities of all chromosomes, which included the two translocated chromosomes.

Data were analyzed using dependent samples *t*-test, general linear model and one-way analysis of variance (or nonparametric tests when data were not normally distributed and the error variance was unequal). A Student–Newman–Keuls, Steel–Dwass post-test or Bonferroni method was used for further comparison when there was statistical significance between groups. All tests were conducted using SPSS version 22.0 (IBM Corp., Armonk, NY, United States). A *p* value < 0.05 was considered significant.

## 3 Results

A total of 261,103 spermatozoa from 10 der (13;14) translocation carriers were analyzed, of which 20,828 were analyzed for translocated chromosomes, including 17,931 haploid, 1,755 nullisomic, 1,024 disomic, 64 diploid, and 54 “others.” A total of 240,275 spermatozoa were analyzed for nontranslocated chromosomes, including 233,483 haploid, 3,184 nullisomic, 2,126 disomic, 1,369 diploid, and 113 “others” (Supplementary Table S4).

### 3.1 Numerical abnormalities per translocated chromosome (13 and 14)

The frequency of nullisomy 13, nullisomy 14, disomy 13, and disomy 14, resulting from adjacent segregation with an average frequency of 23.42% ± 11.63% (range, 13.38%–35.02%) (Table 1), was 7.89% ± 5.62%, 7.04% ± 3.07%, 4.46% ± 1.32%, and 4.03% ± 1.62%, respectively (Supplementary Table S5). The frequency of nullisomy 13 and 14 was 0.97% ± 0.55%, and the frequency of disomy 13 and 14 was 0.66% ± 0.44% (Supplementary Table S5), both resulting from 3:0 segregation with an average frequency of 1.63% ± 0.90% (range, 0.10%–3.08%) (Table 1). Taken together, the frequency of nullisomy 13 was 8.86 ± 5.85%, significantly higher than that of disomy 13 (5.12% ± 1.42%) (*p* < 0.001), and the frequency of nullisomy 14 was 8.01% ± 3.23%, significantly higher than that of disomy 14 (4.69% ± 1.59%) (*p* < 0.001) (Table 1). Overall, the aneuploidy rates of chromosomes 13 and 14 were 13.99% ± 5.80% and 12.71% ± 4.09%, respectively (Table 1).

**TABLE 1:** The analysis of segregation modes and numerical abnormality for translocated chromosomes 13 and 14.

**Patient no.**	**Segregation Modes**					Numerical abnormality							
	**Alternate (%)**	**Adjacent (%)**	**3:00 (%)**	**Diploidy (%)**	**Others (%)**	**Nullisomy 13 (%)**	**Disomy 13 (%)**	**Aneuploidy 13 (%)**	**Total numerical abnormality for chromosome 13 (%)**	**Nullisomy 14 (%)**	**Disomy 14 (%)**	**Aneuploidy 14 (%)**	**Total numerical abnormality for chromosome 14 (%)**
11	62.39	35.02	1.34	0.67	0.57	22.01	3.54	25.55	26.79	8.42	3.73	12.15	13.40
12	62.03	34.19	3.08	0.50	0.20	17.30	6.16	23.46	24.16	12.82	4.08	16.90	17.59
13	75.73	22.59	1.31	0.37	0.00	10.78	4.12	14.90	15.28	4.97	5.34	10.31	10.68
14	78.04	18.69	2.27	0.49	0.49	6.92	3.26	10.19	11.18	5.84	7.22	13.06	14.05
15	79.28	18.30	2.06	0.00	0.36	7.17	6.01	13.18	13.54	5.11	4.13	9.24	9.60
16	71.29	26.43	1.81	0.19	0.29	6.37	6.84	13.21	13.69	10.65	6.18	16.83	17.30
17	85.53	13.38	0.69	0.40	0.00	3.27	3.07	6.34	6.74	6.24	2.18	8.42	8.82
18	65.89	32.95	0.87	0.10	0.19	6.57	6.96	13.53	13.82	14.11	7.05	21.16	21.45
19	80.24	16.22	2.80	0.37	0.37	4.75	6.06	10.81	11.56	7.27	3.73	11.00	11.74
20	83.32	16.48	0.10	0.00	0.10	3.50	5.19	8.69	8.79	4.70	3.30	7.99	8.09
Mean	74.37	23.42	1.63	0.31	0.26	8.86	5.12	13.99	14.55	8.01	4.69	12.71	13.27
SD	8.10	11.63	0.90	0.22	0.19	5.85	1.42	5.80	6.00	3.23	1.59	4.09	4.14

The frequency of diploidy of chromosomes 13 and 14 was 0.31% ± 0.22%, which was not significantly different from that in normozoospermic men (*p* = 0.349) (Table 1; Supplementary Table S5). The incidence of “other” numerical abnormalities was 0.26% ± 0.19%, which was significantly higher than that in normozoospermic men (*p* < 0.01) (Table 1 and Supplementary Table S5). The frequency of total numerical abnormalities for chromosomes 13 and 14 was 14.55% ± 6.00% and 13.27% ± 4.14%, respectively, which was significantly higher than that in normozoospermic men (*p* < 0.001; *p* < 0.001) (Table 1).

Moreover, the mean frequency of disomy 18 was 1.78% ± 1.34% in sperm with an unbalanced number of chromosomes 13 and 14, which was significantly higher than that of in sperm with a balanced number of chromosomes 13 and 14 (0.21% ± 0.30%) (*p* = 0.006) (Supplementary Table S6).

### 3.2 Numerical abnormalities per non-translocated chromosome (1–12, 15–22, X, and Y)

**FIGURE 1:**
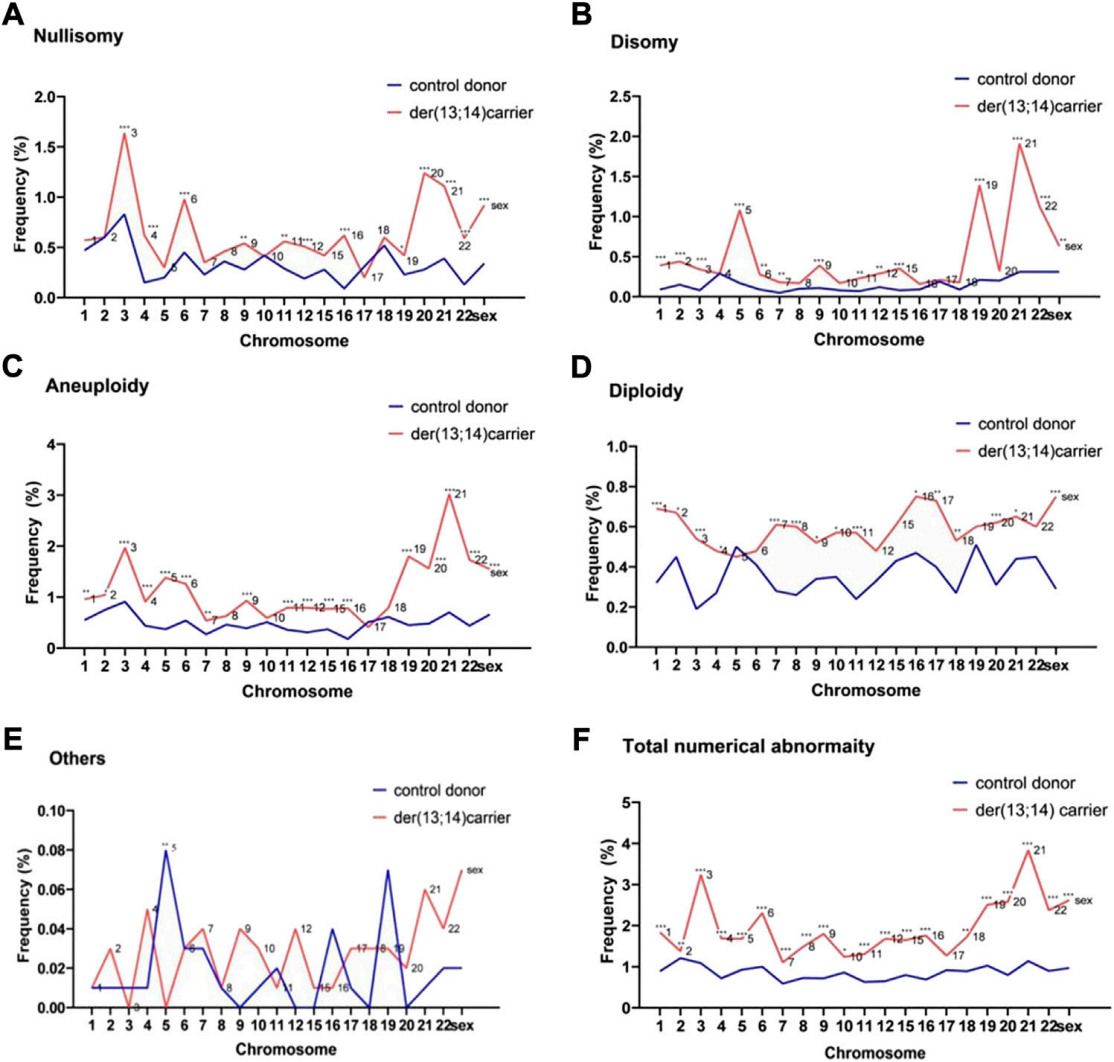
The frequencies of numerical abnormalities of non-translocated chromosomes in sperm of der (13;14) translocation carriers and normozoospermic controls **(A)**. Nullisomy **(B)**. Disomy **(C)**. Aneuploidy (nullisomy + disomy) **(D)**. Diploidy **(E)**. Others **(F)**. Total abnormalities (nullisomy + disomy + diploidy + others). (*p < 0.05; **p < 0.01; ***p < 0.001).

The frequency of nullisomy of the nontranslocated chromosomes in der (13;14) translocation carriers ranged from 0.20% to 1.64%, with an average frequency of 0.65% ± 0.34%, which was significantly higher than that in normozoospermic men (Table 2 and Figure 1A). Specifically, a significant increase was observed in the frequency of nullisomy 3, 4, 6, 9, 11, 12, 16, 19–22, X, and Y compared with that of normozoospermic men, while no significant difference was observed in the frequency of nullisomy 1, 2, 5, 7, 8, 10, 15, 17, and 18 compared with that of normozoospermic men (Table 2 and Figure 1A).

**TABLE 2:** The frequency of numerical abnormalities for nontranslocated chromosomes and the results of comparison with donor controls.

Type of abnormality	Chromosome											
	1	2	3	4	5	6	7	8	9	10	11	12
Nullisomy	0.57	0.6	1.64***	0.62***	0.3	0.98***	0.35	0.46	0.54**	0.41	0.56**	0.51***
Disomy	0.39***	0.44***	0.34***	0.29	1.08***	0.28**	0.18**	0.17	0.39***	0.17	0.23**	0.29**
Diploidy	0.69***	0.67*	0.54***	0.48*	0.45	0.48	0.61***	0.6***	0.52*	0.57*	0.57***	0.48
Others	0.01	0.03	0	0.05	0.00**	0.03	0.04	0.01	0.04	0.03	0.01	0.04
Aneuploidy	0.96**	1.04*	1.97***	0.91***	1.38***	1.26***	0.54**	0.63	0.93***	0.59	0.79***	0.79***
Total numerical abnormality	1.66***	1.74**	2.51***	1.44***	1.83***	1.77***	1.19***	1.25***	1.48***	1.18*	1.38***	1.31***

	**15**	**16**	**17**	**18**	**19**	**20**	**21**	**22**	**SEX**	**Mean**	**SD**	
Nullisomy	0.42	0.62***	0.2	0.61	0.42*	1.24***	1.11***	0.59***	0.92***	0.65***	0.34	
Disomy	0.35***	0.16	0.21	0.18	1.39***	0.32	1.91***	1.13***	0.63**	0.5***	0.46	
Diploidy	0.61	0.75*	0.73**	0.53**	0.6	0.62***	0.65*	0.6	0.75***	0.59***	0.09	
Others	0.01	0.01	0.03	0.03	0.03	0.02	0.06	0.04	0.07	0.03	0.02	
Aneuploidy	0.77***	0.78***	0.41	0.79	1.8***	1.56***	3.02***	1.73***	1.55***	1.15***	0.6	
Total numerical abnormality	1.39***	1.54***	1.16	1.35**	2.43***	2.19***	3.73***	2.37***	2.36***	1.77***	0.62	

**p < 0.05; **p < 0.01; ***p < 0.001.

The frequency of disomy of the nontranslocated chromosomes in der (13;14) translocation carriers ranged from 0.16% to 1.91%, with an average frequency of 0.50% ± 0.46%, which was significantly higher than that in normozoospermic men (Table 2 and Figure 1B). The frequency of disomy 1–3, 5–7, 9, 11-12, 15, 19, 21, 22, X, and Y was significantly higher than that in normozoospermic men, while the frequency of disomy 4, 8, 10, 16–18, and 20 was not significantly different from that in normozoospermic men (Table 2 and Figure 1B). The frequency of aneuploidy (nullisomy + disomy) ranged from 0.41% to 3.02%, with an average frequency of 1.15% ± 0.60%. A significant increase was observed in the frequency of aneuploidy of all non-translocated chromosomes, except chromosomes 8, 10, 17, and 18, when compared with that in normozoospermic men (Table 2 and Figure 1C).

The average frequency of diploidy of the nontranslocated chromosomes was 0.59% ± 0.09%, which was significantly higher than that in normozoospermic men (Table 2 and Figure 1D). The frequency of diploidy of chromosomes 1–4, 7–11, 16–18, 20, 21, X, and Y was significantly higher than that in normozoospermic men, while there was no significant difference in the frequency of diploidy of chromosomes 5, 6, 9, 12, 15, 19, and 22 (Table 2 and Figure 1D).

For the nontranslocated chromosomes in der (13;14) translocation carriers, the average frequency of “others” numerical abnormalities was 0.03% ± 0.02%, with no significant difference from normozoospermic men (Table 2 and Figure 1E). The frequency of total numerical abnormalities was 1.77% ± 0.62% (range, 1.16%–3.73%) (Table 2 and Figure 1F). A significant increase was observed in the frequency of total numerical abnormalities for all nontranslocated chromosomes, except chromosome 17, when compared with that in normozoospermic men (Table 2 and Figure 1F).

**FIGURE 2:**
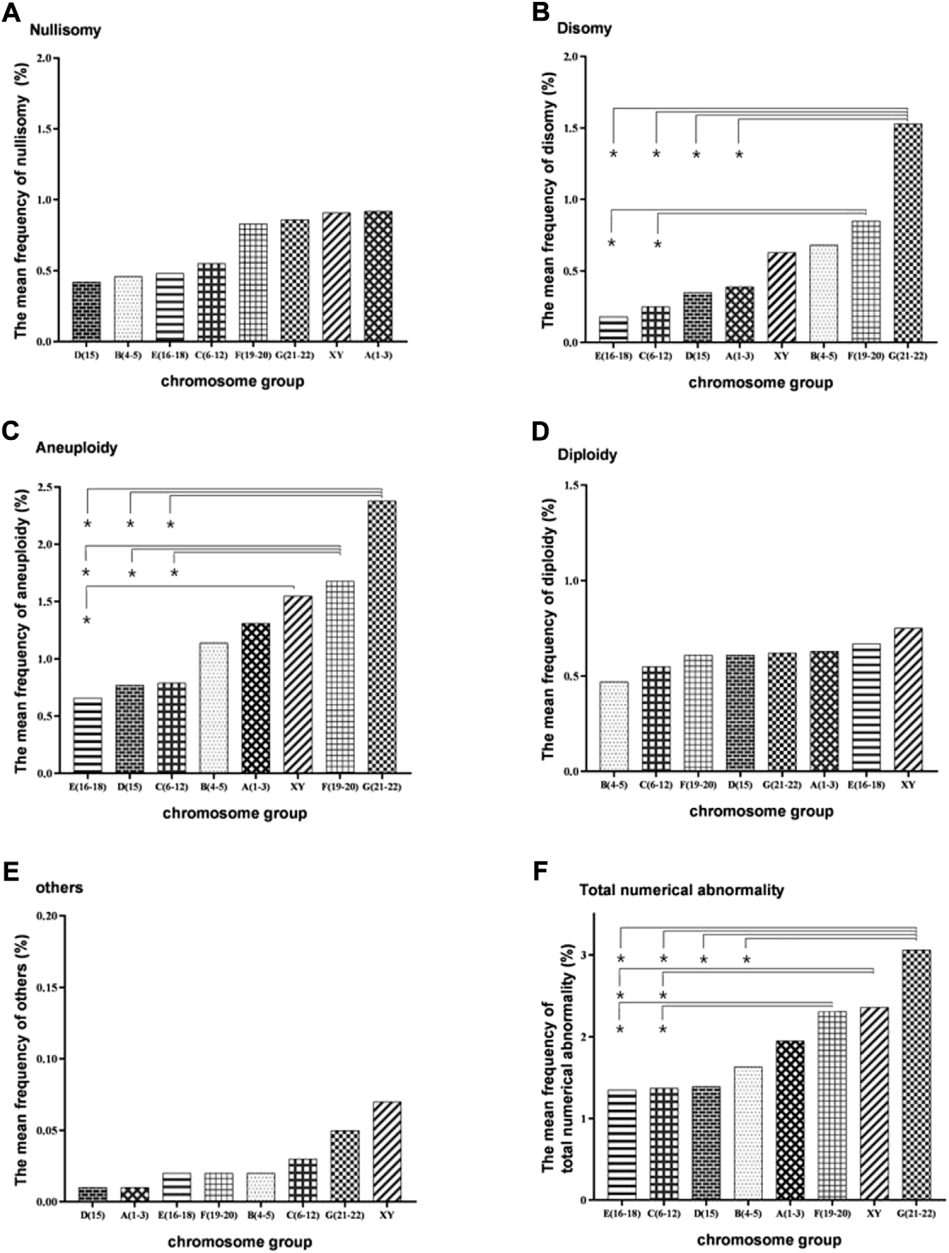
Bar plot of the mean frequencies of nullisomy, disomy, diploidy, others, aneuploidy, and total numerical abnormality in eight subgroups divided by chromosome size. **(A)** Nullisomy **(B)** Disomy **(C)**. Aneuploidy (nullisomy + disomy) **(D)** Diploidy. **(E)** Others **(F)**. Total abnormalities (nullisomy + disomy + diploidy + others). *p < 0.05 (the nonparametric tests was used to calculate the test statistic for all pairwise comparisons of the eight groups to determine whether the frequencies between each pair of groups were significantly different).

Evaluated by chromosome size, the frequency of nullisomy did not differ significantly between the groups A (1–3), B (4–5), C (6–12), D (15), E (16–18), F (19–20), G (21–22), X, and Y (Supplementary Table S7, and Figure 2A). The frequency of disomy in the G (21–22) group was significantly higher than that in the groups A (1–3), C (6–12), D (15), and E (16–18) (Supplementary Table S7, and Figure 2B). The frequency of disomy in the F (19–20) group was significantly higher than that in the groups C (6–12) and E (16–18) (Supplementary Table S7, and Figure 2B). The frequency of aneuploidy in G (21–22) and F (19–20) groups was significantly higher than that in the groups C (6–12) and E (16–18) (Supplementary Table S7, and Figure 2C). The frequency of aneuploidy in X and Y groups was significantly higher than that in the E (16–18) group (Supplementary Table S7, and Figure 2C). The frequency of diploidy and“others” numerical abnormalities did not differ significantly between the groups (Supplementary Table S7, Figure 2D, and Figure 2E). Finally, the frequency of total numerical abnormalities in the G (21–22) group was significantly higher than that in the groups B (4–5), C (6–12), and E (16–18) (Supplementary Table S7, and Figure 2F). The frequency of total numerical abnormalities in the F (19–20) and X and Y groups was significantly higher than that in the groups C (6–12) and E (16-18). (Supplementary Table S7, and Figure 2F). Moreover, in the X and Y group, the frequency of Y-containing sperm in normal haploid spermatozoa was 48.16%, and the frequency of X-containing sperm was 49.48%, with no significant difference between them (Supplementary Table S8). The frequency of XY disomy was significantly higher than that of XX and YY disomy, with an average frequency of 0.31%, 0.16%, and 0.16%, respectively (Supplementary Table S8).

**FIGURE 3:**
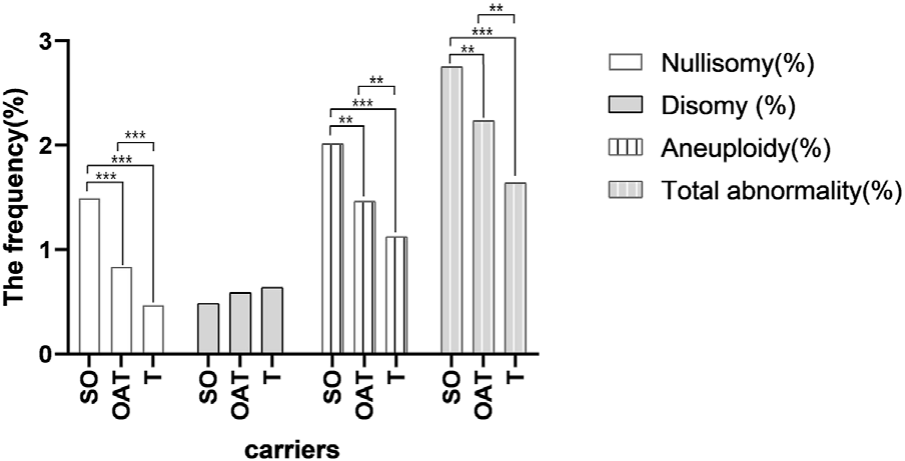
Bar plot of the mean frequencies of nullisomy, disomy, aneuploidy, and total numerical abnormality in three groups of carriers with the different severity of the spermatogenesis. *p < 0.05 **p < 0.01,***p < 0.001(General linear model was conducted to compare the frequency of nullisomy, disomy, diploidy, others, aneuploidy, and total numerical abnormality across different semen groups after controling for the chromosome. The bonferroni method was further used to determine whether the frequencies between each pair of groups were significantly different).

Evaluated by the severity of the spermatogenesis, the mean frequency of non-translocated chromosomes nullisomy in the severe oligozoospermia, oligoasthenoterazoospermia, and teratozoospermia groups were 1.49%, 0.83%, and 0.47%, respectively, with a significant difference between any two groups (*p* < 0.001; *p* < 0.001; *p* < 0.001) (Supplementary Table S9 and Figure 3). The mean frequency of non-translocated chromosome disomy in the severe oligozoospermia, oligoasthenoterazoospermia, and teratozoospermia groups were 0.48%, 0.59%, and 0.64%, respectively, and there was no significant difference between any two of them (*p* = 0.471; *p* = 0.258;*p* = 0.581) (Supplementary Table S9 and Figure 3). The mean frequency of diploidy in the teratozoospermia groups was 0.51%, significant lower than that in the oligoasthenoterazoospermia and severe oligozoospermia group having a mean frequency of 0.77% and 0.74%, respectively (*p* < 0.001;*p* = 0.003) (Supplementary Table S9). The mean frequency of “others” non-translocated chromosome abnormality in the teratozoospermia group was significant lower than that in the oligoasthenoterazoospermia and that in the severe oligozoospermia group, with a mean frequency of 0.02% ± 0.01%, 0.04 ± 0.01 and 0.04% ± 0.01%, respectively (*p* = 0.015) (Supplementary Table S9). The incidence of non-translocation chromosome aneuploidy were significant different in the groups of severe oligozoospermia, oligoasthenoterazoospermia and teratozoospermia (*p* = 0.010; *p* < 0.00; *p* = 0.010), with a mean frequency of 2.01%, 1.46% and 1.13%, respectively (Supplementary Table S9 and Figure 3). The incidence of total non-translocation chromosome abnormality was significant different between each two groups of severe oligozoospermia, oligoasthenoterazoospermia and teratozoospermia (*p* = 0.014; *p* < 0.000; *p* < 0.000), with a mean frequency of 2.75%, 2.23% and 1.64%, respectively (Supplementary Table S9 and Figure 3).

### 3.3 Total cumulative numerical abnormalities per carrier

**FIGURE 4:**
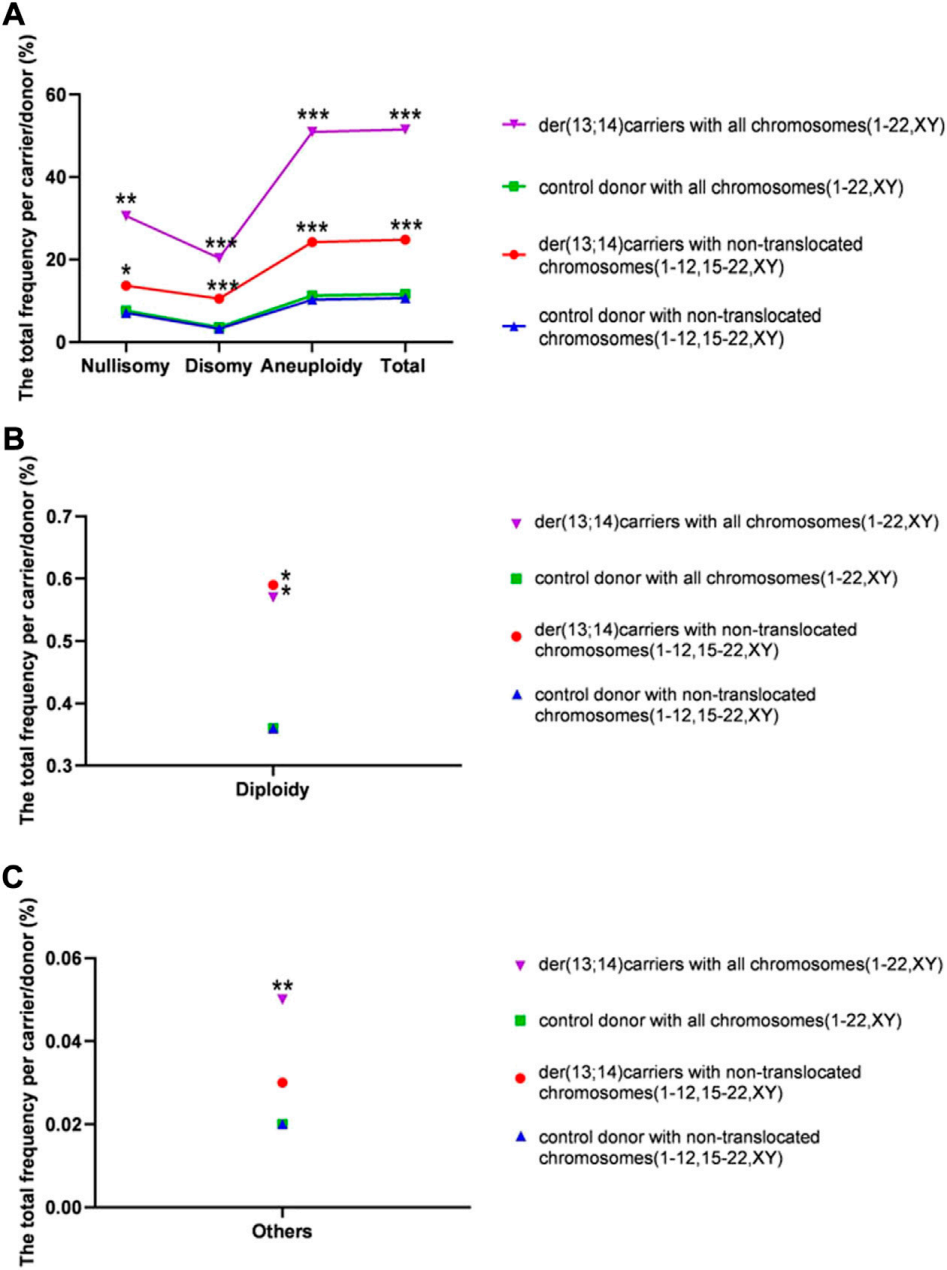
The rates of total numerical abnormalities (including and excluding translocated chromosomes) per carrier in sperm of der (13;14) translocation carriers and per donor in normozoospermic controls. **(A)** The total frequency of nullisomy, disomy, aneuploidy, and total numerical abnormality **(B)**. The frequency of diploidy per carrier **(C)**. The frequency of “others” per carrier. (*p < 0.05; **p < 0.01; ***p < 0.001)

The overall frequency of nullisomy, disomy, and aneuploidy per carrier with nontranslocated chromosomes was 13.69% ± 8.40%, 10.54% ± 4.08%, and 24.22% ± 8.68%, respectively (Table 3 and Figure 4A). The overall frequency of diploidy and “others” per carrier with nontranslocated chromosomes was 0.59% ± 0.23% and 0.03% ± 0.02%, respectively (Table 3 and Figures 4B,C). The frequency of total numerical abnormalities per carrier with nontranslocated chromosomes was 24.84% ± 8.65% (Table 3 and Figure 4A). There was a significant increase in the frequency of all numerical abnormalities, except “others” (*p* = 0.227), per carrier with nontranslocated chromosomes, when compared to that in normozoospermic men (*p* = 0.039; *p* < 0.001; *p* = 0.015; *p* < 0.001; *p* < 0.001) (Table 4 and Figure 4).

**TABLE 3:** The frequency of total nullisomy, disomy, diploidy, others, and aneuploidy, and numerical abnormality per carrier with all chromosomes and with non-translocated chromosomes.

**Type of abnormality**	**With all chromosomes (1–22, X and Y)**			**With nontranslocated chromosomes (1–12,15–22, X and Y)**		
	**der (13;14) translocation carrier**	**Control donor**	***p*-value**	**der (13;14) translocation carrier**	**Control donor**	***p*-value**
Nullisomy	30.57 ± 14.40	7.62 ± 3.23	0.001	13.69 ± 8.40	7.08 ± 3.03	0.039
Disomy	20.35 ± 5.95	3.63 ± 1.18	0.000	10.54 ± 4.08	3.21 ± 0.91	0.000
Diploidy	0.57 ± 0.22	0.36 ± 0.15	0.025	0.59 ± 0.23	0.36 ± 0.15	0.015
Others	0.05 ± 0.02	0.02 ± 0.01	0.006	0.03 ± 0.02	0.02 ± 0.01	0.227
Aneuploidy	50.92 ± 15.40	11.25 ± 3.21	0.000	24.22 ± 8.68	10.29 ± 2.96	0.000
Total	51.53 ± 15.45	11.63 ± 3.19	0.000	24.84 ± 8.65	10.67 ± 2.93	0.000

The overall frequency of nullisomy, disomy, and aneuploidy per carrier with all chromosomes was 30.57% ± 14.40%, 20.35% ± 5.95%, and 50.92% ± 15.40%, respectively (Table 3 and Figure 4A). The overall frequency of diploidy and “others” per carrier with all chromosomes was 0.57% ± 0.22% and 0.05 ± 0.02%, respectively (Table 4 and Figures 4B,C). The frequency of total numerical abnormalities per carrier with all chromosomes was 51.53% ± 15.45% (Table 3 and Figure 4A). There was a significant increase in the frequency of all numerical abnormalities per carrier with translocated chromosomes when compared to that in normozoospermic men (*p* = 0.001; *p* < 0.001; *p* = 0.025; *p* = 0.006; *p* < 0.001; *p* < 0.001) (Table 3 and Figure 4).

## 4 Discussion

While the segregation of translocated chromosomes and the ICE on some non-translocated chromosomes in Robertsonian translocation der (13;14) (q10;q10) carriers has been investigated in several studies, only a few studies have analyzed the numerical abnormalities of all chromosomes. The aim of this study was to analyze the frequency of haploidy, nullisomy, disomy, aneuploidy, diploidy, “others,” and the total numerical abnormalities of all chromosomes (1–22, X, and Y), thereby, revealing more specifically and objectively how much translocated chromosome segregation errors and the ICE of nontranslocated chromosomes can lead to abnormal chromosome numbers in sperm.

### 4.1 Numerical abnormality of translocated chromosomes

Many researchers have analyzed the segregation modes in spermatozoa of der (13;14) translocation carriers, reporting 20.7% unbalanced gametes, of which 19.7% ± 10.2% were from adjacent, 0.8% ± 1.0% from 3:0, and 0.2% ± 0.2% from “others” segregation modes, respectively (Wiland et al., 2020). The results in this study were consistent with those of previous studies (Supplementary Figure S2), with mean frequencies of 23.42% ± 11.63%, 1.63% ± 0.90%, and 0.26% ± 0.19% for adjacent, 3:0 and “others,” respectively. Abnormal numbers of chromosomes 13 and 14 are mostly due to adjacent segregation, with Lamotte et al. (2018) reporting a specific frequency of 5.83% ± 3.00% for disomy 13, 6.69% ± 3.44% for nullisomy 13, 5.76% ± 2.60% for disomy 14, and 6.59% ± 2.72% for nullisomy 14 from adjacent segregation, which was consistent with the results of this study (4.46% ± 1.32% for disomy 13, 7.89% ± 5.62% for nullisomy 13, 4.03% ± 1.62% for disomy 14, and 7.04% ± 3.07% for nullisomy 14). In addition, we differentiated diploid sperm from 3:0 segregated sperm using a tri-color probe set, and found that approximately 16% of 3:0 segregated sperm were diploid. The average frequency of 3:0 segregated sperm, after removal of this fraction of diploid sperm, was 1.63%. Finally, by further adding 3:0 and “others,” the total frequencies of numerical anomalies for chromosomes 13 and 14 were calculated to be 14.55% ± 6.00% and 13.27% ± 4.14%, respectively, indicating that the interference of derived chromosomes during the segregation of translocation chromosomes was significant.

In addition, sperm with balanced numbers of chromosomes 13 and 14 could be distinguished from sperm with unbalanced numbers of chromosomes 13 and 14 based on the number of fluorescent signals. If other nontranslocated chromosome-specific probes were added to the probe mixture of chromosomes 13 and 14, it was possible to assess whether spermatozoa with an unbalanced number of translocated chromosomes had more chromosomal imbalance than spermatozoa with a balanced number of translocated chromosomes. Our results showed that sperm with an unbalanced number of chromosomes 13 and 14 have a significant higher rate of disomy 18 than sperm with a balanced number of chromosomes 13 and 14. Unfortunately, our data on whether spermatozoa with an unbalanced number of translocated chromosomes had more chromosomal imbalance than spermatozoa with a balanced number of translocated chromosomes were very limited and confined to chromosome 18. Nonetheless, it requires comprehensive studies on this aspect, including more nontranslocated chromosomes.

### 4.2 Numerical abnormality of non-translocated chromosomes

The frequency of numerical abnormalities of nontranslocated chromosomes has also been reported. A review of 15 FISH studies on the ICE in Robertsonian translocation carriers found 292 records of disomy and nullisomy for 14 chromosomes (1–3, 7–9, 12, 15, 17, 18, 21, 22, X, and Y) in 63 der (13;14) translocation carriers (Supplementary Table S10). Of these, 150 records were the disomy frequencies for chromosomes 18, 21, X, and Y, which had higher survival rates in neonates. The average frequency of disomy chromosome 21 and disomy sex chromosomes was 1.19% and 0.71%, respectively, consistent with the results of this study (1.91% and 0.63%, respectively), while the average frequency of disomy chromosome 18 was 0.43%, which was higher than that obtained in this study (0.18%). The average frequency of disomy for chromosome 22 reported by 22 records was one third of that in the present study (0.34% vs. 1.13%). The disomy frequencies for other chromosomes were scarcely reported; there were only 41 records for 9 chromosomes (1–3, 7–9, 12, 15, and 17), of which the frequency of disomy 3, 8 and 12 was 0.41%, 0.18%, and 0.35%, similar to that in the present study (0.34%, 0.17%, and 0.29%, respectively), the frequency of disomy 2, 7 and 9 was 0.18%, 0.08%, and 0.16%, approximately one-fold lower than that in the present study (0.44%, 0.18%, and 0.39%), and the frequency of disomy 1 and 15 was 1.39% and 1.37%, approximately two-fold higher than that in the present study (0.39% and 0.35%, respectively). Some studies analyzed the incidence of nullisomy for chromosomes 18, 21, 22, and the sex chromosomes with an anverage frequency of 0.73%, 2.09%, 0.32%, and 1.12%, respectively, which was similar to that in this study (0.61%, 1.11%, 0.59%, and 0.92%, respectively). In addition, the average frequency of diploidy was 0.60%, which was also consistent with the results of this study (0.59%). Aside from all the investigated chromosomes, the disomy frequencies for eight other chromosomes (4–6, 10, 11, 19, and 20) and the nullisomy frequencies for 17 other chromosomes (1–12, 15–17, 19, and 20) were reported in this study. These frequencies have not been reported earlier (Supplementary Figure S3), providing limited data to supplement those in the current study in understanding numerical chromosomal abnormalities from a more specific and nuanced perspective. Therefore, further validation is required.

Machev et al*.* (2005) suggested that the size of the chromosome could significantly increase the rate of disomy, with longer chromosomes being more prone to nondisjunction. To further analyze the size-dependent variation in numerical chromosomal abnormalities, the 22 non-translocated chromosomes were divided into eight groups, namely, A (1–3), B (4–5), C (6–12), D (15), E (16–18), F (19–20), G (21–22), X and Y, according to size. We found that the frequency of disomy was significantly higher in the G (21–22) group than in the groups A(1–3), C(6–12), D(15), and E(16-18) and that the frequency of disomy in the F (19–20) group was significantly higher than that in the C (6–12) and E (16–18) groups, suggesting that smaller chromosomes are more prone to nondisjunction than the bigger chromosomes.

Some studies showed that numerical abnormality could correlated with the semen parameter (Shi and Martin, 2001; Calogero et al., 2003; Vegetti et al., 2000), therefore, the frequency of nullisomy, disomy, diploidy, aneuploidy, others and total abnormality were assessed by the severity of the spermatogenesis. However, there were only ten carriers in the study with semen analysis of 1 severe oligozoospermia, 3 oligoasthenoteratozoospermia, and 6 teratozoospermia, the number of carriers in each group was small, and it was difficult to carry out the variation of numerical abnormalities in perspective of per carrier. Thus, we analyzed differences of abnormality by taking all nontranslocated chromosomes as a whole in the analysis. Interestingly, there was a significant difference in the frequency of non-translocation chromosome nullisomy in each two of the three groups (teratozoospermia, oligoasthenoteratozoospermia and severe oligozoospermia). The severe oligozoospermia group had the highest nullisomy rate followed by that in the oligoasthenoteratozoospermia group and that in the teratozoospermia group, which indicated that the nullisomy rate of non-translocated chromosomes increased with the severity of sperm. In contrast, the mean incidence of non-translocation chromosomal disomy decreased with the severity of abnormal spermatogenesis, with the highest disomy rate in the teratozoospermia group followed by that in the oligoasthenoteratozoospermia group and that in the severe oligozoospermia group. However, the differences in non-translocation chromosome disomy rates among the groups did not reach a significant level. The frequency of aneuploidy and total numerical abnormality for non-translocated chromosomes increased with sperm severity, both showing the highest rate in the severe oligozoospermia followed by that in the oligoasthenoteratozoospermia and that in teratozoospermia. Nonetheless, the results requires further evaluation by analyzing the numerical abnormality in sperm of more der (13;14) translocation male carriers.

### 4.3 The interchromosomal effect (ICE)

To evaluate the presence of the ICE, numerical abnormalities of each of the 22 nontranslocated chromosomes were compared with those of normozoospermic men. The frequency of disomy for 15 chromosomes (1–3, 5–7, 9, 11, 12, 15, 19, 21, 22, X, and Y) was significantly higher than that in normozoospermic men, with the highest frequency observed for disomy 21, followed by that for 19, 22, 5, the sex chromosomes, and chromosomes 2, 9, 1, 15, 3, 20, 12, 4, 6, and 11. Our results are consistent with those of previous studies analyzing the frequency of disomy for chromosomes 18, 21, X, and Y, all of which showed a significant increase in the frequency of disomy for the sex chromosomes (Baccetti et al., 2005; Godo et al., 2015; Wang et al., 2017) and chromosome 21 (Mahjoub et al., 2011; Vozdova et al., 2013; Hajlaoui et al., 2018) and a nonsignificant increase in the frequency of disomy for chromosome 18 (Machev et al., 2005; Douet-guilbert et al., 2005; Baccetti et al., 2005; Ogur et al., 2006; Chen et al., 2007; Kekesi et al., 2007; Wang et al., 2017; Olszewska et al., 2021).

The frequency of nullisomy for 13 chromosomes (3, 4, 6, 9, 11, 12, 16, 19–22, X, and Y) was significantly higher than that in normozoospermic men. Our results are consistent with those of previous studies in which significantly higher frequencies of sex chromosome nullisomy were observed (Ogur et al., 2006; Wang et al., 2017) and nullisomy 22 (Hajlaoui et al., 2018) and inconsistent with those of previous studies that reported significantly higher frequency of nullisomy 18 (Mahjoub et al., 2011; Vozdova et al., 2013; Hajlaoui et al., 2018). The aneuploidy rate was significantly higher than that in normozoospermic men, for all chromosomes, except for chromosomes 10, 17, and 18. The frequency of diploidy for 15 chromosomes (1–4, 7–11, 17, 18, 20, 21, X, and Y) was significantly higher than that in normozoospermic men. However, no significant differences were observed in the frequency of “other” numerical abnormalities. The total numerical abnormalities per chromosome were significantly higher than that in normozoospermic men, except for chromosome 17, indicating the presence of the ICE in der (13;14) translocation carriers. Furthermore, a significant difference was observed in the total cumulative frequencies of nullisomy, disomy, diploidy, and the total numerical abnormalities between each der (13;14) translocation carrier and normozoospermic man, further indicating the presence of the ICE.

The mechanism of the ICE is speculated to involve unpaired regions of the short arms of normal chromosomes in a trivalent structure that triggers the formation of heterosynapses between the trivalent structure and some chromosomes, such as the sex and acrocentric chromosomes, bearing homologous blocks, to enable meiosis to proceed. The formation of heterosynapses may interfere with the normal segregation of the corresponding chromosomes, and of other chromosome pairs, resulting in a high incidence of nullisomy and disomy. Our results show that the frequency of nullisomy and disomy for the sex and acrocentric chromosomes (e.g., 21 and 22), as well as for several other chromosomes, was significantly increased, further supporting this hypothesis. Although the abnormal incidence of each nontranslocated chromosome was lower than that of the translocated chromosomes, the overall abnormal incidence of non-translocated chromosomes for each carrier was comparable to that of the translocated chromosomes due to the large number of chromosomes.

### 4.4 Future directions and limitations

In this study, the chromosome-specific variation in numerical abnormality was comprehensively with a strong statistical power that was supported by the large number of appromiately 10,000 spermatozoa analyzed for each of the 22 autosomes and 2 sex chromosomes. The assessment of carrier-specific variation in numerical abnormalities was not statistically supported by ten der (13;14) translocation carriers, although the number of ten male der (13;14) translocation carriers was not small in the study regarding Robertsonian translations due to the low incidence of der (13;14) translocation male carriers (Supplementary Table S10), which is a limitation of this study. Furthermore, we could not find any normozoospermic carriers, with all carriers analyzed presenting abnormal semen parameters. While, in some studies, the numerical abnormality correlated with the semen parameter (Vegetti et al., 2000; Shi and Martin, 2001; Calogero et al., 2003), raising concerns that the increase above normozoospemic males might be, to some extent, attributed to the abnormal semen parameter. Therefore, more carriers with normal and abnormal sperm should be analyzed in the future to assess whether there is any increased fraction that is not from ICE and to evaluate the carrier-specific variation in numerical abnormalities.

In conclusion, numerical abnormalities were observed in the sperm of der (13;14) translocation carriers, with a high incidence of abnormalities in the translocated chromosomes (13 and 14), and a significantly higher incidence of abnormalities in the nontranslocated chromosomes compared with that in normozoospermic men. Despite the small number of carriers (*n* = 10) and controls (*n* = 10) that make carrier-based analyses difficult to support statistically, the statistical analyses performed on a per-sperm basis were reliable. These findings suggest that the intervention of translocation-derived chromosome in the meiosis was significant, involving both translocated and nontranslocated chromosomes.

## Data Availability

The raw data supporting the conclusion of this article will be made available by the authors, without undue reservation.

## References

[B1] Anton E. Blanco J. Egozcue J. Vidal F. (2004). Sperm FISH studies in seven male carriers of Robertsonian translocation t(13;14)(q10;q10). Hum. Reprod. 19 (6), 1345–1351. 10.1093/humrep/deh232 15117905

[B2] Baccetti B. Collodel G. Marzella R. Moretti E. Piomboni P. Scapigliati G. (2005). Ultrastructural studies of spermatozoa from infertile males with Robertsonian translocations and 18, X, Y aneuploidies. Hum. Reprod. 20 (8), 2295–2300. 10.1093/humrep/dei050 15878922

[B3] Balasar O. Acar H. (2020). Investigation of the interchromosomal effects in male carriers with structural chromosomal abnormalities using FISH. Turk. J. Urol. 46 (3), 178–185. 10.5152/tud.2020.19255 32301691 PMC7219968

[B4] Brugnon F. Van Assche E. Verheyen G. Sion B. Boucher D. Pouly J. L. (2006). Study of two markers of apoptosis and meiotic segregation in ejaculated sperm of chromosomal translocation carrier patients. Hum. Reprod. 21 (3), 685–693. 10.1093/humrep/dei401 16339168

[B5] Calogero A. E. Burrello N. De Palma A. Barone N. D'Agata R. Vicari E. (2003). Sperm aneuploidy in infertile men. Reprod. Biomed. Online 6 (3), 310–317. 10.1016/s1472-6483(10)61850-0 12735865

[B6] Cassuto N. G. Le Foll N. Chantot-Bastaraud S. Balet R. Bouret D. Rouen A. (2011). Sperm fluorescence *in situ* hybridization study in nine men carrying a robertsonian or a reciprocal translocation: Relationship between segregation modes and high-magnification sperm morphology examination. Fertil. Steril. 96 (4), 826–832. 10.1016/j.fertnstert.2011.07.1143 21871621

[B7] Chen Y. Huang J. Liu P. Qiao J. (2007). Analysis of meiotic segregation patterns and interchromosomal effects in sperm from six males with Robertsonian translocations. J. Assist. Reprod. Genet. 24 (9), 406–411. 10.1007/s10815-007-9137-6 17653848 PMC3454947

[B8] Cooper T. G. Noonan E. von Eckardstein S. Auger J. Baker H. W. Behre H. M. (2010). World Health Organization reference values for human semen characteristics. Hum. Reprod. Update 16 (3), 231–245. 10.1093/humupd/dmp048 19934213

[B9] Douet-guilbert N. Bris M. J. L. Amice V. Marchetti C. Delobel B. Amice J. (2005). Interchromosomal effect in sperm of males with translocations: Report of 6 cases and review of the literature. Int. J. Androl. 28 (6), 372–379. 10.1111/j.1365-2605.2005.00571.x 16300670

[B10] Escudero T. Lee M. Carrel D. Blanco J. Munne S. (2000). Analysis of chromosome abnormalities in sperm and embryos from two 45, XY, t(13;14)(q10;q10) carriers. Prenat. Diagn. 20 (7), 599–602. 10.1002/1097-0223(200007)20:7<599::aid-pd883>3.3.co;2-h 10913961

[B11] Ferfouri F. Selva J. Boitrelle F. Gomes D. M. Torre A. Albert M. (2011). The chromosomal risk in sperm from heterozygous Robertsonian translocation carriers is related to the sperm count and the translocation type. Fertil. Steril. 96 (6), 1337–1343. 10.1016/j.fertnstert.2011.09.008 21963229

[B12] Frydman N. Romana S. Le Lorc'H M. Vekemans M. Frydman R. Tachdjian G. (2001). Assisting reproduction of infertile men carrying a Robertsonian translocation. Hum. Reprod. 16 (11), 2274–2277. 10.1093/humrep/16.11.2274 11679503

[B13] Godo A. Blanco J. Vidal F. Sandalinas M. Garcia-Guixe E. Anton E. (2015). Altered segregation pattern and numerical chromosome abnormalities interrelate in spermatozoa from Robertsonian translocation carriers. Reprod. Biomed. Online. 31 (1), 79–88. 10.1016/j.rbmo.2015.04.003 25985997

[B14] Hajlaoui A. Slimani W. Kammoun M. Sallem A. Braham S. Bibi M. (2018). Sperm fluorescent *in situ* hybridisation study of interchromosomal effect in six Tunisian carriers of reciprocal and Robertsonian translocations. Andrologia 50 (4), e12949. 10.1111/and.12949 29336050

[B15] Kekesi A. Erdei E. Torok M. Dravucz S. Toth A. (2007). Segregation of chromosomes in spermatozoa of four Hungarian translocation carriers. Fertil. Steril. 88 (1), e5–11. 10.1016/j.fertnstert.2006.11.097 17274993

[B16] Lamotte A. Martinez G. Devillard F. Hograindleur J. Satre V. Coutton C. (2018). Is sperm FISH analysis still useful for robertsonian translocations? Meiotic analysis for 23 patients and review of the literature. Basic Clin. Androl. 28 (1), 5. 10.1186/s12610-018-0069-z 29760927 PMC5937048

[B17] Lejeune J. (1963). Autosomal disorders. Pediatrics 32, 326–337. 10.1542/peds.32.3.326 14063510

[B18] Machev N. Gosset P. Warter S. Treger M. Schillinger M. Viville S. (2005). Fluorescence *in situ* hybridization sperm analysis of six translocation carriers provides evidence of an interchromosomal effect. Fertil. Steril. 84 (2), 365–373. 10.1016/j.fertnstert.2005.03.026 16084877

[B19] Mahjoub M. Mehdi M. Brahem S. Elghezal H. Ibala S. Saad A. (2011). Chromosomal segregation in spermatozoa of five Robertsonian translocation carriers t(13;14). J. Assist. Reprod. Genet. 28 (7), 607–613. 10.1007/s10815-011-9560-6 21448573 PMC3162055

[B20] Miller D. E. (2020). The interchromosomal effect: Different meanings for different organisms. Genetics 216 (3), 621–631. 10.1534/genetics.120.303656 33158985 PMC7648586

[B21] Morel F. Roux C. Bresson J. L. (2001). FISH analysis of the chromosomal status of spermatozoa from three men with 45, XY, der(13;14)(q10;q10) karyotype. Mol. Hum. Reprod. 7 (5), 483–488. 10.1093/molehr/7.5.483 11331672

[B22] Ogur G. Van Assche E. Vegetti W. Verheyen G. Tournaye H. Bonduelle M. (2006). Chromosomal segregation in spermatozoa of 14 Robertsonian translocation carriers. Mol. Hum. Reprod. 12 (3), 209–215. 10.1093/molehr/gah253 16524928

[B23] Olszewska M. Wiland E. Wanowska E. Huleyuk N. Chernykh V. B. Zastavna D. (2021). Analysis of sperm chromosomes in six carriers of rare and common Robertsonian translocations^*^ . Postep. Hig. Med. Dosw. 75, 199–210. 10.5604/01.3001.0014.8122

[B24] Perrin A. Nguyen M. H. Bujan L. Vialard F. Amice V. Gueganic N. (2013). DNA fragmentation is higher in spermatozoa with chromosomally unbalanced content in men with a structural chromosomal rearrangement. Andrology 1 (4), 632–638. 10.1111/j.2047-2927.2013.00100.x 23785022

[B25] Poot M. Hochstenbach R. (2021). Prevalence and phenotypic impact of robertsonian translocations. Mol. Syndromol. 12 (1), 1–11. 10.1159/000512676 33776621 PMC7983559

[B26] Pylyp L. Y. Zukin V. D. Bilko N. M. (2013). Chromosomal segregation in sperm of Robertsonian translocation carriers. J. Assist. Reprod. Genet. 30 (9), 1141–1145. 10.1007/s10815-013-0067-1 23893157 PMC3800540

[B27] Rouen A. Pyram K. Pollet-Villard X. Hyon C. Dorna M. Marques S. (2013). Simultaneous cell by cell study of both DNA fragmentation and chromosomal segregation in spermatozoa from chromosomal rearrangement carriers. J. Assist. Reprod. Genet. 30 (3), 383–390. 10.1007/s10815-012-9915-7 23288665 PMC3607690

[B28] Shi Q. Martin R. H. (2001). Aneuploidy in human spermatozoa: FISH analysis in men with constitutional chromosomal abnormalities, and in infertile men. Reproduction 121 (5), 655–666. 10.1530/rep.0.1210655 11427153

[B29] Therman E. Susman B. Denniston C. (1989). The nonrandom participation of human acrocentric chromosomes in Robertsonian translocations. Ann. Hum. Genet. 53 (1), 49–65. 10.1111/j.1469-1809.1989.tb01121.x 2658738

[B30] Vegetti W. Van Assche E. Frias A. Verheyen G. Bianchi M. M. Bonduelle M. (2000). Correlation between semen parameters and sperm aneuploidy rates investigated by fluorescence *in-situ* hybridization in infertile men. Hum. Reprod. 15 (2), 351–365. 10.1093/humrep/15.2.351 10655307

[B31] Vozdova M. Oracova E. Kasikova K. Prinosilova P. Rybar R. Horinova V. (2013). Balanced chromosomal translocations in men: Relationships among semen parameters, chromatin integrity, sperm meiotic segregation and aneuploidy. J. Assist. Reprod. Genet. 30 (3), 391–405. 10.1007/s10815-012-9921-9 23318982 PMC3607677

[B32] Wang B. Nie B. Tang D. Li R. Liu X. Song J. (2017). Analysis of meiotic segregation patterns and interchromosomal effects in sperm from 13 robertsonian translocations. Balk. J. Med. Genet. 20 (1), 43–50. 10.1515/bjmg-2017-0003 PMC5596821 28924540

[B33] Wiland E. Olszewska M. Wozniak T. Kurpisz M. (2020). How much, if anything, do we know about sperm chromosomes of Robertsonian translocation carriers? Cell. Mol. Life Sci. 77 (23), 4765–4785. 10.1007/s00018-020-03560-5 32514588 PMC7658086

