# Supplementary material for: FISH analysis of numerical chromosomal abnormalities in the sperm of robertsonian translocation der(13; 14)(q10;q10) carriers
Source: Front Genet. 2022 Sep 27;13:1010568. doi: 10.3389/fgene.2022.1010568 (PMC9551382; doi:10.3389/fgene.2022.1010568)
Supplement: Supplementary file 1 [file Table1.DOCX]

| **Supplementary Table 1.** Numerical abnormalities of disomy and nullisomy for pairs of translocated chromosomes in previous studies | | | | | | | | | |
| --- | --- | --- | --- | --- | --- | --- | --- | --- | --- |
| **Reference** | **Patient** | **Alternate (%)** | **Adjacent (%)** | | | | **3:00 (%)** | **“Others” (%)** | **Unbalanced total (%)** |
|  |  |  | Disomy 13 | Nullisomy 13 | Disomy 14 | Nullisomy 14 |  |  |  |
| Lamotte *et al*.^3^ | 1 | 72.76 | 6.34 | 8.21 | 2.99 | 6.72 | 2.99 | – | 27.24 |
|  | 2 | 71.69 | 4.57 | 6.39 | 7.31 | 10.05 | 0.00 | – | 28.31 |
|  | 3 | 64.94 | 4.60 | 15.52 | 8.62 | 5.75 | 0.57 | – | 35.06 |
|  | 4 | 84.86 | 2.83 | 4.66 | 3.16 | 4.49 | 0.00 | – | 15.14 |
|  | 5 | 85.95 | 2.42 | 5.82 | 2.75 | 3.07 | 0.00 | – | 14.05 |
|  | 6 | 75.39 | 6.84 | 6.29 | 5.74 | 5.52 | 0.22 | – | 24.61 |
|  | 7 | 78.52 | 7.21 | 6.38 | 3.52 | 4.36 | 0.00 | – | 21.48 |
|  | 8 | 66.08 | 4.59 | 3.81 | 9.97 | 12.12 | 3.42 | – | 33.92 |
|  | 9 | 60.69 | 13.08 | 3.15 | 7.75 | 7.27 | 8.06 | – | 39.31 |
|  | Mean±SD | 73.43±8.23 | 5.83±3.00 | 6.69±3.44 | 5.76±2.60 | 6.59±2.72 | 1.70±2.58 | – | 26.57±8.23 |
| Pylyp *et al*.^19^ | 1 | 78.40 | 4.80 | 6.90 | 3.20 | 5.40 | 1.40 | – | 21.70 |
|  | 2 | 75.50 | 6.10 | 4.40 | 4.10 | 5.30 | 2.00 | – | 21.90 |
|  | 3 | 81.20 | 2.90 | 3.60 | 3.80 | 4.20 | 3.70 | – | 18.20 |
|  | 4 | 86.50 | 4.30 | 1.70 | 3.10 | 3.20 | 1.10 | – | 13.40 |
|  | 5 | 69.40 | 5.90 | 4.40 | 7.30 | 8.60 | 4.20 | – | 30.40 |
|  | Mean±SD | 78.20±5.70 | 4.80±1.16 | 4.20±1.67 | 4.30±1.55 | 5.34±1.82 | 2.48±1.24 | – | 21.12±5.57 |
| Mahjoub *et al*.^10^ | 1 | – | 8.05 | 9.40 | 8.72 | 7.38 | – | – | – |
|  | 2 | – | 8.11 | 4.32 | 3.78 | 4.86 | – | – | – |
|  | 3 | – | 7.93 | 4.87 | 6.10 | 3.05 | – | – | – |
|  | 4 | – | 7.82 | 9.50 | 8.94 | 6.70 | – | – | – |
|  | 5 | – | 8.48 | 7.27 | 5.45 | 7.88 | – | – | – |
|  | Mean±SD | 0.00±0.00 | 8.08±0.22 | 7.07±2.18 | 6.60±1.97 | 5.97±1.78 | – | – | – |
| Anton *et al*.^15^ | 1 | 86.48 | 2.87 | 4.04 | 2.28 | 3.38 | 0.44 | 0.51 | – |
|  | 2 | 87.49 | 2.96 | 4.21 | 2.10 | 2.90 | 0.00 | 0.34 | – |
|  | 3 | 83.00 | 1.19 | 3.06 | 0.40 | 9.88 | 0.20 | 2.27 | – |
|  | 4 | 84.53 | 2.50 | 6.19 | 0.99 | 4.49 | 0.20 | 1.10 | – |
|  | 5 | 88.13 | 1.57 | 5.11 | 1.81 | 2.91 | 0.39 | 0.08 | – |
|  | 6 | 88.23 | 2.37 | 4.36 | 2.38 | 2.01 | 0.31 | 0.34 | – |
|  | 7 | 87.73 | 3.62 | 2.45 | 1.94 | 3.62 | 0.39 | 0.26 | – |
|  | Mean±SD | 86.51±1.86 | 2.44±0.77 | 4.20±1.15 | 1.70±0.68 | 4.17±2.43 | 0.28±0.14 | 0.70±0.71 | – |
| Frydman *et al*.^17^ | 1 | 91.00 | 2.00 | 2.00 | 3.50 | 1.50 | – | – | 9.00 |
|  | 2 | 90.00 | 1.40 | 2.60 | 4.40 | 1.60 | – | – | 10.00 |
|  | 3 | 87.10 | 3.30 | 3.30 | 3.50 | 2.80 | – | – | 12.90 |
|  | Mean±SD | 89.37±1.65 | 2.23±0.79 | 2.63±0.53 | 3.80±0.42 | 1.97±0.59 | – | – | 10.63±1.65 |
| Escudero *et al*.^18^ | 1 | 73.60 | 5.00 | 4.70 | 4.10 | 9.50 | – | 3.10 | 26.40 |
|  | 2 | 77.40 | 3.70 | 4.40 | 4.20 | 6.80 | – | 3.40 | 22.60 |
|  | Mean±SD | 75.50±1.90 | 4.35±0.65 | 4.55±0.15 | 4.15±0.05 | 8.15±1.35 | – | 3.25±0.15 | 24.50±1.90 |
| Total Mean±SD | | 79.87±8.36 | 4.82±2.65 | 5.26±2.69 | 4.45±2.47 | 5.40±2.67 | 1.41±1.99 | 1.27±1.23 | 22.40±8.49 |

­SD, standard deviation.

| **Supplementary Table 2.** Semen parameters of the 10 Robertsonian translocation der(13;14)(q10;q10) carriers and controls | | | | | | | |
| --- | --- | --- | --- | --- | --- | --- | --- |
| **Group** | **Sample** | **Age (years)** | **Volume (mL)** | **Concentration (10^6^/mL)** | **Progressive motility, PR%(a+b)** | **Normal morphology (%)** | **Seminogram** |
| Controls | 1 | 30 | 2.0 | 164 | 62 | >4 | normozoospermia |
|  | 2 | 29 | 3.5 | 109 | 60 | >4 | normozoospermia |
|  | 3 | 28 | 3.0 | 76 | 62 | >4 | normozoospermia |
|  | 4 | 36 | 4.7 | 76 | 64 | >4 | normozoospermia |
|  | 5 | 25 | 6.7 | 70 | 60 | >4 | normozoospermia |
|  | 6 | 25 | 3.0 | 150 | 60 | >4 | normozoospermia |
|  | 7 | 37 | 2.5 | 111 | 60 | >4 | normozoospermia |
|  | 8 | 39 | 4.7 | 79 | 61 | >4 | normozoospermia |
|  | 9 | 29 | 2.7 | 68 | 62 | >4 | normozoospermia |
|  | 10 | 29 | 2.5 | 60 | 63 | >4 | normozoospermia |
|  | Mean±SD | 30.7±4.7 | 3.5±1.4 | 96.3±34.3 | 61.4±1.4 | – | – |
| der(13;14) carriers | 11 | 34 | 0.8 | 14.1 | 20.7 | 1.5 | oligoasthenoteratozoospermia |
|  | 12 | 30 | 1.2 | 0–1 motile sperm/4–5 HP, 1–2 sperm/HP | | | oligozoospermia |
|  | 13 | 33 | 3.2 | 52.9 | 50.9 | 2.5 | teratozoospermia |
|  | 14 | 33 | 2.0 | 7.9 | 16.1 | 0.5 | oligoasthenoteratozoospermia |
|  | 15 | 37 | 4.9 | 31.3 | 64.8 | 2.0 | teratozoospermia |
|  | 16 | 34 | 3.8 | 99.6 | 62.2 | 3.5 | teratozoospermia |
|  | 17 | 28 | 3.0 | 44.4 | 46.7 | 3.0 | teratozoospermia |
|  | 18 | 32 | 1.1 | 5.6 | 5.7 | 0.5 | oligoasthenoteratozoospermia |
|  | 19 | 38 | 3.1 | 31.8 | 60.2 | 2.0 | teratozoospermia |
|  | 20 | 31 | 2.1 | 18.2 | 52.3 | 2.5 | teratozoospermia |
|  | Mean±SD | 33.0±2.9 | 2.5±1.2 | 34.0±27.7 | 42.2±20.8 | 2.0±1.0 | – |
| WHO reference values | | – | 1.5 | 15 | 32 | 4 | – |
| The WHO reference values refer to the fifth edition of the WHO Laboratory Manual for the Examination and Processing of Human Semen (2010).  SD, standard deviation; WHO, World Health Organization. | | | | | | | |

| **Supplementary Table 3**. Mix of probes used for FISH analyses | | | | | | |
| --- | --- | --- | --- | --- | --- | --- |
| For donors | | |  | For carriers | | |
| Chromosomes | Assessed probe | Control probe |  | Chromosomes | Assessed probe | Control probe |
| 1 | CEP1 (SO) | CEP X (SG) / CEPY (SA) |  | 1 | CEP1 (SO) | CEP X (SG) / CEPY (SA) |
| 2 | CEP2 (SO) | CEP X (SG) / CEPY (SA) |  | 2 | CEP2 (SO) | CEP X (SG) / CEPY (SA) |
| 3 | CEP3 (SO) | CEP X (SG) / CEPY (SA) |  | 3 | CEP3 (SO) | CEP X (SG) / CEPY (SA) |
| 4 | CEP4 (SA) | CEP X (SG) / CEPY (SO) |  | 4 | CEP4 (SA) | CEP X (SG) / CEPY (SO) |
| 5 | TEL 5q (SO) | CEP 18 (SA) |  | 5 | TEL 5q (SO) | CEP 18 (SA) |
| 6 | CEP 6 (SA) | CEP X (SG) / CEPY (SO) |  | 6 | CEP 6 (SA) | CEP X (SG) / CEPY (SO) |
| 7 | CEP 7 (SA) | CEP X (SG) / CEPY (SO) |  | 7 | CEP 7 (SA) | CEP X (SG) / CEPY (SO) |
| 8 | CEP 8 (SA) | CEP X (SG) / CEPY (SO) |  | 8 | CEP 8 (SA) | CEP X (SG) / CEPY (SO) |
| 9 | CEP 9 (SA) | CEP X (SG) / CEPY (SO) |  | 9 | CEP 9 (SA) | CEP X (SG) / CEPY (SO) |
| 10 | CEP 10 (SA) | CEP X (SG) / CEPY (SO) |  | 10 | CEP 10 (SA) | CEP X (SG) / CEPY (SO) |
| 11 | CEP 11 (SA) | CEP X (SG) / CEPY (SO) |  | 11 | CEP 11 (SA) | CEP X (SG) / CEPY (SO) |
| 12 | CEP 12 (SG) | CEP 18 (SA) |  | 12 | CEP 12 (SG) | CEP 18 (SA) |
| 13 | RB1 (SO) | CEP 18 (SA) |  | 13/14 | RB1 (SG)/TEL 14q (SO) | CEP 18 (SA) |
| 14 | TEL 14q (SO) | CEP 18 (SA) |  |  |  |  |
| 15 | TEL 15q (SO) | CEP 18 (SA) |  | 15 | TEL 15q (SO) | CEP 18 (SA) |
| 16 | CEP 16 (SA) | CEP X (SG) / CEPY (SO) |  | 16 | CEP 16 (SA) | CEP X (SG) / CEPY (SO) |
| 17 | CEP 17 (SA) | CEP X (SG) / CEPY (SO) |  | 17 | CEP 17 (SA) | CEP X (SG) / CEPY (SO) |
| 18 | CEP 18 (SA) | CEP X (SG) / CEPY (SO) |  | 18 | CEP 18 (SA) | CEP X (SG) / CEPY (SO) |
| 19 | TEL 19q (SO) | CEP 18 (SA) |  | 19 | TEL 19q (SO) | CEP 18 (SA) |
| 20 | TEL 20q (SO) | CEP 18 (SA) |  | 20 | TEL 20q (SO) | CEP 18 (SA) |
| 21 | LSI 21 (SO) | CEP 18 (SA) |  | 21 | LSI 21 (SO) | CEP 18 (SA) |
| 22 | TEL 22q (SO) | CEP 18 (SA) |  | 22 | TEL 22q (SO) | CEP 18 (SA) |

| **Supplementary Table 4.** Distribution of numerical chromosomal abnormalities in Robertsonian translocation der(13;14)(q10;q10) carriers and controls | | | | | | | | | | | | | | | |
| --- | --- | --- | --- | --- | --- | --- | --- | --- | --- | --- | --- | --- | --- | --- | --- |
| **Group** | **22 nontranslocated chromosomes** | | | | | | |  | **Translocated chromosomes 13 and 14** | | | | | |  |
|  | **Sample** | **Haploidy n (%)** | **Nullisomy**  **n (%)** | **Disomy n (%)** | **Diploidy n (%)** | **“Others”**  **n (%)** | **Total** |  | **Haploidy n (%)** | **Nullisomy n (%)** | **Disomy n (%)** | **Diploidy n (%)** | **“Others” n (%)** | **Total** | **Total** |
| Controls | 1 | 23,043 (99.40) | 57 (0.25) | 35 (0.02) | 42 (0.18) | 4 (0.02) | 23,181 |  | 2,149 (99.22) | 10 (0.46) | 6 (0.28) | 1 (0.05) | 0 (0.00) | 2166 | 25347 |
|  | 2 | 22,491 (99.26) | 65 (0.29) | 33 (0.01) | 67 (0.30) | 3 (0.01) | 22,659 |  | 2,093 (99.52) | 2 (0.10) | 2 (0.10) | 4 (0.19) | 2 (0.10) | 2103 | 24762 |
|  | 3 | 22,289 (98.83) | 96 (0.43) | 39 (0.02) | 127 (0.56) | 3 (0.01) | 22554 |  | 1,944 (98.53) | 6 (0.30) | 5 (0.25) | 15 (0.76) | 3 (0.15) | 1973 | 24527 |
|  | 4 | 22,108 (99.03) | 72 (0.32) | 43 (0.02) | 100 (0.45) | 2 (0.01) | 22,325 |  | 1,996 (98.81) | 6 (0.30) | 4 (0.20) | 12 (0.59) | 2 (0.10) | 2020 | 24345 |
|  | 5 | 22,517 (99.12) | 143 (0.63) | 29 (0.01) | 29 (0.13) | 0 (0.00) | 22,718 |  | 2,316 (98.85) | 14 (0.60) | 5 (0.21) | 8 (0.34) | 0 (0.00) | 2343 | 25061 |
|  | 6 | 23,094 (98.92) | 68 (0.29) | 58 (0.02) | 116 (0.50) | 10 (0.04) | 23,346 |  | 2,152 (98.58) | 3 (0.14) | 12 (0.55) | 15 (0.69) | 1 (0.05) | 2183 | 25529 |
|  | 7 | 22,510 (99.03) | 60 (0.26) | 27 (0.01) | 128 (0.56) | 5 (0.02) | 22,730 |  | 2,247 (99.38) | 2 (0.09) | 2 (0.09) | 7 (0.31) | 3 (0.13) | 2261 | 24991 |
|  | 8 | 22,429 (99.10) | 110 (0.49) | 24 (0.01) | 68 (0.30) | 1 (0.00) | 22,632 |  | 2,221 (99.37) | 5 (0.22) | 2 (0.09) | 6 (0.27) | 1 (0.04) | 2235 | 24867 |
|  | 9 | 22,850 (99.35) | 29 (0.13) | 36 (0.02) | 77 (0.33) | 7 (0.03) | 22,999 |  | 2,045 (99.13) | 5 (0.24) | 5 (0.24) | 7 (0.34) | 1 (0.05) | 2063 | 25062 |
|  | 10 | 22,096 (99.35) | 60 (0.27) | 24 (0.01) | 60 (0.27) | 1 (0.00) | 22,241 |  | 2,068 (99.47) | 5 (0.24) | 2 (0.10) | 4 (0.19) | 0 (0.00) | 2079 | 24320 |
|  | total | 225,427 (99.14) | 760 (0.33) | 348 (0.15) | 814 (0.36) | 36 (0.02) | 227,385 |  | 21,231 (99.09) | 58 (0.27) | 45 (0.21) | 79 (0.37) | 13 (0.06) | 21426 | 248811 |
| der (13; 14) carriers | 11 | 23,395 (96.47) | 480 (1.98) | 155 (0.64) | 207 (0.85) | 15 (0.06) | 24,252 |  | 1,670 (79.90) | 318 (15.22) | 76 (3.64) | 14 (0.67) | 12 (0.57) | 2090 | 26342 |
|  | 12 | 22,636 (95.84) | 621 (2.63) | 185 (0.78) | 165 (0.70) | 12 (0.05) | 23,619 |  | 1,592 (79.13) | 303 (15.06) | 103 (5.12) | 10 (0.50) | 4 (0.20) | 2012 | 25631 |
|  | 13 | 24,349 (97.59) | 240 (0.96) | 201 (0.81) | 152 (0.61) | 8 (0.03) | 24,950 |  | 1,857 (87.02) | 168 (7.87) | 101 (4.73) | 8 (0.37) | 0 (0.00) | 2134 | 27084 |
|  | 14 | 23,173 (96.95) | 349 (1.46) | 201 (0.84) | 155 (0.65) | 24 (0.10) | 23,902 |  | 1,767 (87.39) | 129 (6.38) | 106 (5.24) | 10 (0.49) | 10 (0.49) | 2022 | 25924 |
|  | 15 | 23,727 (97.46) | 353 (1.45) | 221 (0.91) | 33 (0.14) | 11 (0.05) | 24,345 |  | 1,972 (88.43) | 137 (6.14) | 113 (5.07) | 0 (0.00) | 8 (0.36) | 2230 | 26575 |
|  | 16 | 23,500 (97.15) | 237 (0.98) | 296 (1.22) | 145 (0.60) | 11 (0.05) | 24,189 |  | 1,778 (84.51) | 179 (8.51) | 137 (6.51) | 4 (0.19) | 6 (0.29) | 2104 | 26293 |
|  | 17 | 23,234 (97.98) | 161 (0.68) | 127 (0.54) | 188 (0.79) | 3 (0.01) | 23,713 |  | 1,861 (92.13) | 96 (4.75) | 53 (2.62) | 8 (0.40) | 2 (0.10) | 2020 | 25733 |
|  | 18 | 23,043 (96.55) | 362 (1.52) | 288 (1.21) | 160 (0.67) | 13 (0.05) | 23,866 |  | 1,705 (82.37) | 214 (10.34) | 145 (7.00) | 2 (0.10) | 4 (0.19) | 2070 | 25936 |
|  | 19 | 23,186 (97.39) | 240 (1.01) | 302 (1.27) | 70 (0.29) | 10 (0.04) | 23,808 |  | 1,896 (88.43) | 129 (6.02) | 105 (4.90) | 8 (0.37) | 6 (0.28) | 2144 | 25952 |
|  | 20 | 23,240 (98.35) | 141 (0.60) | 150 (0.63) | 94 (0.40) | 6 (0.03) | 23,631 |  | 1,833 (91.56) | 82 (4.10) | 85 (4.25) | 0 (0.00) | 2 (0.10) | 2002 | 25633 |
|  | Total | 233,483 (97.17) | 3,184 (1.33) | 2,126 (0.88) | 1,369 (0.57) | 113 (0.05) | 240,275 |  | 17,931 (86.09) | 1,755 (8.43) | 1,024 (4.92) | 64 (0.31) | 54 (0.26) | 20828 | 261103 |

| **Supplementary Table 5.** Meiotic segregation of Robertsonian translocation der(13;14)(q10;q10) carriers | | | | | | | | | | | | |
| --- | --- | --- | --- | --- | --- | --- | --- | --- | --- | --- | --- | --- |
| **Patient** | **Alternate (%)** | **Adjacent (%)** | | | | |  | **3:0 (%)** | | | **Diploidy (%)** | **“Others” (%)** |
|  |  | **Nullisomy 13** | **Disomy 13** | **Nullisomy 14** | **Disomy 14** | **Total** |  | **Nullisomy 13 and 14** | **Disomy 13 and 14** | **Total** |  |  |
| 11 | 62.39 | 20.86 | 3.35 | 7.27 | 3.54 | 35.02 |  | 1.15 | 0.19 | 1.34 | 0.67 | 0.57 |
| 12 | 62.03 | 15.41 | 4.97 | 10.93 | 2.88 | 34.19 |  | 1.89 | 1.19 | 3.08 | 0.50 | 0.20 |
| 13 | 75.73 | 10.12 | 3.47 | 4.31 | 4.69 | 22.59 |  | 0.66 | 0.66 | 1.31 | 0.37 | 0.00 |
| 14 | 78.04 | 5.44 | 2.47 | 4.35 | 6.43 | 18.69 |  | 1.48 | 0.79 | 2.27 | 0.49 | 0.49 |
| 15 | 79.28 | 6.46 | 4.66 | 4.39 | 2.78 | 18.30 |  | 0.72 | 1.35 | 2.06 | 0.00 | 0.36 |
| 16 | 71.29 | 5.23 | 6.18 | 9.51 | 5.51 | 26.43 |  | 1.14 | 0.67 | 1.81 | 0.19 | 0.29 |
| 17 | 85.53 | 2.78 | 2.87 | 5.75 | 1.98 | 13.38 |  | 0.50 | 0.20 | 0.69 | 0.40 | 0.00 |
| 18 | 65.89 | 5.99 | 6.67 | 13.53 | 6.76 | 32.95 |  | 0.58 | 0.29 | 0.87 | 0.10 | 0.19 |
| 19 | 80.24 | 3.17 | 4.85 | 5.68 | 2.52 | 16.22 |  | 1.58 | 1.21 | 2.80 | 0.37 | 0.37 |
| 20 | 83.32 | 3.50 | 5.09 | 4.70 | 3.20 | 16.48 |  | 0.00 | 0.10 | 0.10 | 0.00 | 0.10 |
| Mean±SD | 74.37±8.10 | 7.89±5.62 | 4.46±1.32 | 7.04±3.07 | 4.03±1.62 | 23.42±11.63 |  | 0.97±0.55 | 0.66±0.44 | 1.63±0.90 | 0.31±0.22 | 0.26±0.19 |

SD, standard deviation.

| **Supplementary table 6 \|** The mean frequencies of nullisomy 18, disomy 18 and aneuploidy 18 in sperms with a balanced and unabalanced number of chromosomes 13 and 14 | | | |
| --- | --- | --- | --- |
| **Group of sperm** | **Type of abnormality** | | |
|  | **Nullisomy 18(%)** | **Disomy 18(%)*** | **Aneuploidy 18(%)** |
| 13,14–balance | 0.88±1.59 | 0.21±0.30 | 1.09±1.82 |
| 13,14–imbalance | 1.66±2.51 | 1.78±1.34 | 3.44±3.07 |

*p<0.05

| **Supplementary Table 7 \|** The mean frequencies of nullisomy, disomy, diploidy, and others, aneuploidy, and total numerical abnormality in eight chromosome groups by size. | | | | | | | | | |
| --- | --- | --- | --- | --- | --- | --- | --- | --- | --- |
| **Type of abnormality** | **Chromosome group** | | | | | | | | |
|  | **A(1–3)** | **B(4–5)** | **C(6–12)** | **D(15)** | **E(16–18)** | **F(19–20)** | **G(21–22)** | **XY** | **p–value** |
| Nullisomy (%) | 0.92 | 0.46 | 0.55 | 0.42 | 0.48 | 0.83 | 0.86 | 0.91 | 0.316 |
| Disomy (%) | 0.39 | 0.68 | 0.25 | 0.35 | 0.18 | 0.85 | 1.53 | 0.63 | 0.000 |
| Diploidy (%) | 0.63 | 0.47 | 0.55 | 0.61 | 0.67 | 0.61 | 0.62 | 0.75 | 0.313 |
| Others (%) | 0.01 | 0.02 | 0.03 | 0.01 | 0.02 | 0.02 | 0.05 | 0.07 | 0.133 |
| Aneuploidy (%) | 1.31 | 1.14 | 0.79 | 0.77 | 0.66 | 1.68 | 2.38 | 1.55 | 0.000 |
| Total numerical abnormality (%) | 1.95 | 1.63 | 1.37 | 1.39 | 1.35 | 2.31 | 3.06 | 2.36 | 0.000 |

| **Supplementary Table 8.** Frequency of haploidy, nullisomy, disomy, diploidy, and “others” for pairs of sex chromosomes (X and Y) | | | | | | | | | | | | | |
| --- | --- | --- | --- | --- | --- | --- | --- | --- | --- | --- | --- | --- | --- |
| **Group** | **Patient** | **Chromosome** | **Haploidy** | | | **Nullisomy** | **Disomy** | | | | **Diploidy, n (%)** | **“Others,” n (%)** | **Total** |
|  |  |  | **Y, n (%)** | **X, n (%)** | **Total** |  | **XX, n (%)** | **XY, n (%)** | **YY, n (%)** | **Total** |  |  |  |
| Controls | 1 | 23 | 491 (48.81) | 503 (50.00) | 994 | 1 (0.10) | 0 (0.00) | 6 (0.60) | 1 (0.10) | 7 | 4 (0.40) | 0 (0.00) | 1,006 |
|  | 2 | 23 | 502 (48.74) | 519 (50.39) | 1,021 | 6 (0.58) | 0 (0.00) | 0 (0.00) | 0 (0.00) | 0 | 3 (0.29) | 0 (0.00) | 1,030 |
|  | 3 | 23 | 476 (47.70) | 511 (51.20) | 987 | 0 (0.00) | 3 (0.30) | 0 (0.00) | 0 (0.00) | 3 | 8 (0.80) | 0 (0.00) | 998 |
|  | 4 | 23 | 548 (52.74) | 483 (46.49) | 1,031 | 4 (0.38) | 0 (0.00) | 0 (0.00) | 1 (0.10) | 1 | 3 (0.29) | 0 (0.00) | 1,039 |
|  | 5 | 23 | 554 (50.09) | 542 (49.01) | 1,096 | 3 (0.27) | 0 (0.00) | 6 (0.54) | 0 (0.00) | 6 | 1 (0.09) | 0 (0.00) | 1,106 |
|  | 6 | 23 | 519 (46.63) | 574 (51.57) | 1,093 | 10 (0.90) | 1 (0.09) | 7 (0.63) | 0 (0.00) | 8 | 1 (0.09) | 1 (0.09) | 1,113 |
|  | 7 | 23 | 515 (48.09) | 548 (51.17) | 1,063 | 4 (0.37) | 0 (0.00) | 1 (0.09) | 0 (0.00) | 1 | 3 (0.28) | 0 (0.00) | 1,071 |
|  | 8 | 23 | 501 (47.44) | 552 (52.27) | 1,053 | 1 (0.09) | 0 (0.00) | 1 (0.09) | 0 (0.00) | 1 | 1 (0.09) | 0 (0.00) | 1,056 |
|  | 9 | 23 | 539 (49.36) | 544 (49.82) | 1,083 | 3 (0.27) | 0 (0.00) | 0 (0.00) | 1 (0.09) | 1 | 5 (0.46) | 0 (0.00) | 1,092 |
|  | 10 | 23 | 536 (53.02) | 463 (45.80) | 999 | 4 (0.40) | 3 (0.30) | 1 (0.10) | 1 (0.10) | 5 | 2 (0.20) | 1 (0.10) | 1,011 |
|  | mean | | 518 (49.26) | 524 (49.77) | 1,042 | 4 (0.34) | 1 (0.07) | 2 (0.21) | 0 (0.04) | 3 | 3 (0.30) | 0 (0.02) | 1,052 |
| der (13; 14) carriers | 11 | 23 | 516 (49.47) | 503 (48.23) | 1,019 | 5 (0.48) | 2 (0.19) | 3 (0.29) | 1 (0.10) | 6 | 12 (1.15) | 1 (0.10) | 1,043 |
|  | 12 | 23 | 474 (47.12) | 503 (50.00) | 977 | 15 (1.49) | 1 (0.10) | 2 (0.20) | 2 (0.20) | 5 | 7 (0.70) | 2 (0.20) | 1,006 |
|  | 13 | 23 | 544 (46.70) | 599 (51.42) | 1,143 | 4 (0.34) | 4 (0.34) | 4 (0.34) | 0 (0.00) | 8 | 9 (0.77) | 1 (0.09) | 1,165 |
|  | 14 | 23 | 487 (45.90) | 535 (50.42) | 1,022 | 20 (1.89) | 0 (0.00) | 8 (0.75) | 0 (0.00) | 8 | 9 (0.85) | 2 (0.19) | 1,061 |
|  | 15 | 23 | 554 (48.22) | 568 (49.43) | 1,122 | 19 (1.65) | 0 (0.00) | 6 (0.52) | 0 (0.00) | 6 | 2 (0.17) | 0 (0.00) | 1,149 |
|  | 16 | 23 | 406 (39.76) | 597 (58.47) | 1,003 | 1 (0.10) | 2 (0.20) | 0 (0.00) | 4 (0.39) | 6 | 11 (1.08) | 0 (0.00) | 1,021 |
|  | 17 | 23 | 538 (51.04) | 489 (46.39) | 1,027 | 3 (0.28) | 2 (0.19) | 1 (0.09) | 5 (0.47) | 8 | 16 (1.52) | 0 (0.00) | 1,054 |
|  | 18 | 23 | 497 (47.38) | 527 (50.24) | 1,024 | 10 (0.95) | 2 (0.19) | 6 (0.57) | 2 (0.19) | 10 | 4 (0.38) | 1 (0.10) | 1,049 |
|  | 19 | 23 | 598 (58.57) | 410 (40.16) | 1,008 | 4 (0.39) | 3 (0.29) | 1 (0.10) | 3 (0.29) | 7 | 2 (0.20) | 0 (0.00) | 1,021 |
|  | 20 | 23 | 487 (47.42) | 514 (50.05) | 1,001 | 16 (1.56) | 1 (0.10) | 2 (0.19) | 0 (0.00) | 3 | 7 (0.68) | 0 (0.00) | 1,027 |
|  | Mean | | 510 (48.16) | 525 (49.48) | 1,035 | 10 (0.91) | 2 (0.16) | 3 (0.31) | 2 (0.16) | 7 | 8 (0.75) | 1 (0.07) | 1,060 |

| **Supplementary table 9 \|** The mean frequencies of nullisomy, disomy, diploidy, others, aneuploidy and total numerical abnormality in carriers with different severity of the spermatogenesis. | | | | | | |
| --- | --- | --- | --- | --- | --- | --- |
| **Group of carriers** | **Type of abnormality** | | | | | |
|  | **Nullisomy(%)** | **Disomy (%)** | **Diploidy(%)** | **Others(%)** | **Aneuploidy(%)** | **Total abnormality(%)** |
| Oligoasthenoterazoospermia | 0.83a | 0.59 | 0.77a | 0.04a | 1.46a | 2.23a |
| Severe oligozoospermia | 1.49b | 0.48 | 0.74a | 0.04a,b | 2.01b | 2.75b |
| Teratozoospermia | 0.47c | 0.64 | 0.51b | 0.02b | 1.13c | 1.64c |

| **Supplementary Table 10.** Published data on sperm disomy, nullisomy, and diploidy rates for nontranslocated chromosomes in Robertsonian translocation der(13;14)(q10;q10) carriers | | | | | | | | | | | | | | | | | | | |
| --- | --- | --- | --- | --- | --- | --- | --- | --- | --- | --- | --- | --- | --- | --- | --- | --- | --- | --- | --- |
| **Reference** | **der(13;14) carrier** | **Disomy** | | | | | | | | | | | | | **Diploidy** | **Nullisomy** | | | |
|  |  | **1+** | **2+** | **3+** | **7+** | **8+** | **9+** | **12+** | **15+** | **17+** | **18+** | **21+** | **22+** | **Sex+** |  | **18–** | **21–** | **22–** | **Sex–** |
| Chen *et al*.^28^ | 1 | – | – | – | – | – | – | – | – | – | 0.00 | – | – | 0.67 | 0.19 | 0.29 | – | – | 0.19 |
|  | 2 | – | – | – | – | – | – | – | – | – | 0.09 | – | – | 0.57 | 0.47 | 0.19 | – | – | 2.47 |
|  | 3 | – | – | – | – | – | – | – | – | – | 0.00 | – | – | 4.56 | 6.46 | 0.76 | – | – | 2.66 |
|  | 4 | – | – | – | – | – | – | – | – | – | 0.00 | – | – | 0.29 | 3.01 | 0.14 | – | – | 0.57 |
| Baccetti *et al*.^27^ | 1 | – | – | – | – | – | – | – | – | – | 0.16 | – | – | 0.16 | 0.67 | – | – | – | – |
|  | 2 | – | – | – | – | – | – | – | – | – | 0.23 | – | – | 0.58 | 0.58 | – | – | – | – |
|  | 3 | – | – | – | – | – | – | – | – | – | 0.14 | – | – | 0.66 | 1.09 | – | – | – | – |
|  | 4 | – | – | – | – | – | – | – | – | – | 0.05 | – | – | 0.29 | 0.67 | – | – | – | – |
| Hajlaoui *et al*.^21^ | 5 | – | – | – | – | – | – | – | – | – | 2.49 | 1.92 | 2.50 | – | 0.96 | 1.92 | – | 1.34 | 0.57 |
|  | 6 | – | – | – | – | – | – | – | – | – | 1.77 | 4.74 | 2.96 | – | 0.88 | 0.98 | – | 1.38 | 0.00 |
| Machev *et al*.^29^ | 2 | 0.33 | – | – | – | – | – | – | 0.87 |  | 0.35 | – | – | – | 0.03 | – | – | – | – |
|  | 3 | 1.63 | – | – | – | – | – | – | 1.87 |  | 0.21 | – | – | – | 0.18 | – | – | – | – |
|  | 4 | 2.21 | – | – | – | – | – | – | – | 0.50 | – | – | – | – | 0.03 | – | – | – | – |
| Balasar and Acar^24^ | 2 | – | 0.16 | 0.21 | – | – | – | 0.49 | – | 0.42 | 1.48* | 0.20 | – | – | – | – | – | – | – |
|  | 10 | – | 0.20 | 0.60 | – | – | – | 0.20 | – | 0.40 | 0.22 | 0.49 | – | – | – | – | – | – | – |
| Douet-Guilbert *et al*.^31^ | 1 | – | – | – | 0.10 | – | 0.08 | – | – | – | 0.17 | 0.16 | – | – | 0.23 | – | – | – | – |
| Olszewska *et al*.^25^ | 4 | – | – | – | 0.13 | – | 0.13 | – | – | – | 0.07 | 0.08 | 0.16 | – | 0.24 | – | – | – | – |
|  | 5 | – | – | – | – | – | – | – | – | – | 0.00 | 0.00 | 0.00 | – | 0.00 | – | – | – | – |
|  | 6 | – | – | – | 0.32 | – | 0.26 | – | – | – | 0.12 | 0.00 | 0.00 | – | 0.06 | – | – | – | – |
| Kékesi *et al*.^16^ | 3 | – | – | – | – | – | – | – | – | – | 0.20 | – | – | – | 0.30 | – | – | – | – |
| Morel *et al*.^30^ | 1 | – | – | – | – | 1.21 | – | – | – | – | 0.77 | 0.80 | – | – | 0.83 | – | – | – | – |
|  | 2 | – | – | – | – | 0.50 | – | – | – | – | 0.78 | 0.49 | – | – | 0.88 | – | – | – | – |
|  | 3 | – | – | – | – | 0.21 | – | – | – | – | 0.30 | 0.40 | – | – | 0.10 | – | – | – | – |
| Vozdova *et al*.^5^ | 1 | – | – | – | 0.01 | 0.00 | – | – | – | – | 0.03 | 0.05 | – | 0.08 | 0.08 | – | – | – | – |
|  | 2 | – | – | – | 0.04 | 0.02 | – | – | – | – | 0.04 | 0.23 | – | 0.29 | 0.22 | – | – | – | – |
|  | 3 | – | – | – | – | 0.04 | – | – | – | – | 0.03 | 0.21 | – | 0.23 | 0.22 | – | – | – | – |
|  | 5 | – | – | – | – | 0.04 | – | – | – | – | 0.12 | 0.11 | – | 0.16 | 1.28 | – | – | – | – |
|  | 6 | – | – | – | 0.02 | 0.04 | – | – | – | – | 0.11 | 0.33 | – | 0.60 | 0.45 | – | – | – | – |
|  | 7 | – | – | – | 0.03 | 0.03 | – | – | – | – | 0.06 | 0.37 | – | 0.15 | 0.55 | – | – | – | – |
|  | 8 | – | – | – | – | 0.13 | – | – | – | – | 0.19 | 0.34 | – | 1.75 | 1.63 | – | – | – | – |
|  | 9 | – | – | – | 0.08 | 0.08 | – | – | – | – | 0.10 | 0.09 | – | 0.17 | 0.31 | – | – | – | – |
|  | 10 | – | – | – | – | 0.02 | – | – | – | – | 0.05 | 0.26 | – | 0.26 | 0.12 | – | – | – | – |
|  | 11 | – | – | – | 0.04 | 0.11 | – | – | – | – | 0.06 | 0.35 | – | 0.51 | 0.22 | – | – | – | – |
|  | 12 | – | – | – | 0.02 | 0.03 | – | – | – | – | 0.03 | 0.21 | – | 0.29 | 0.31 | – | – | – | – |
| Godo *et al*.^2^ | 1 | – | – | – | – | – | – | – | – | – | 0.15 | 0.01 | 0.05 | 0.22 | 0.40 | 0.04 | 0.16 | 0.04 | 0.40 |
|  | 2 | – | – | – | – | – | – | – | – | – | 0.05 | 0.08 | 0.03 | 0.40 | 0.12 | 0.04 | 0.08 | 0.02 | 0.67 |
|  | 3 | – | – | – | – | – | – | – | – | – | 0.22 | 0.04 | 0.01 | 0.47 | 0.23 | 0.11 | 0.24 | 0.16 | 0.74 |
|  | 4 | – | – | – | – | – | – | – | – | – | 0.11 | 0.14 | 0.10 | 0.18 | 1.46 | 0.05 | 0.36 | 0.14 | 0.58 |
|  | 5 | – | – | – | – | – | – | – | – | – | 0.17 | 0.09 | 0.02 | 1.21 | 0.38 | 0.45 | 0.16 | 0.36 | 1.31 |
|  | 6 | – | – | – | – | – | – | – | – | – | 0.09 | 0.05 | 0.07 | 0.40 | 0.15 | 0.18 | 0.04 | 0.02 | 0.45 |
|  | 7 | – | – | – | – | – | – | – | – | – | 0.15 | 0.77 | 0.33 | 0.52 | 0.71 | 0.14 | 0.08 | 0.02 | 0.26 |
|  | 8 | – | – | – | – | – | – | – | – | – | 0.15 | 0.08 | 0.24 | 0.49 | 0.41 | 0.05 | 0.11 | 0.01 | 0.28 |
|  | 9 | – | – | – | – | – | – | – | – | – | 0.11 | 0.08 | 0.08 | 0.75 | 0.48 | 0.20 | 0.23 | 0.09 | 0.91 |
|  | 10 | – | – | – | – | – | – | – | – | – | 0.10 | 0.08 | 0.03 | 0.46 | 0.31 | 0.03 | 0.13 | 0.22 | 0.62 |
| Wang *et al*.^4^ | 3 | – | – | – | – | – | – | – | – | – | 0.50 | – | – | 2.10 | – | 0.60 | – | – | 2.20 |
|  | 4 | – | – | – | – | – | – | – | – | – | 0.50 | – | – | 1.20 | – | 0.40 | – | – | 1.70 |
|  | 5 | – | – | – | – | – | – | – | – | – | 0.90 | – | – | 1.50 | – | 1.10 | – | – | 1.30 |
|  | 6 | – | – | – | – | – | – | – | – | – | 0.20 | – | – | 1.70 | – | 0.70 | – | – | 1.50 |
|  | 7 | – | – | – | – | – | – | – | – | – | 1.20 | – | – | 0.50 | – | 0.90 | – | – | 1.00 |
|  | 8 | – | – | – | – | – | – | – | – | – | 1.30 | – | – | 1.90 | – | 2.10 | – | – | 2.40 |
| Anton *et al*.^15^ | 1 | – | – | – | – | – | – | – | – | – | 0.00 | 0.11 | 0.00 | 0.23 | 0.25 | – | – | – | – |
|  | 2 | – | – | – | – | – | – | – | – | – | 0.11 | 0.10 | 0.10 | 0.44 | 0.03 | – | – | – | – |
|  | 3 | – | – | – | – | – | – | – | – | – | 0.12 | 0.07 | 0.07 | 0.94 | 0.10 | – | – | – | – |
|  | 4 | – | – | – | – | – | – | – | – | – | 0.00 | 0.06 | 0.17 | 0.18 | 0.18 | – | – | – | – |
|  | 5 | – | – | – | – | – | – | – | – | – | 0.05 | 0.31 | 0.18 | 0.47 | 0.42 | – | – | – | – |
|  | 6 | – | – | – | – | – | – | – | – | – | 0.00 | 0.66 | 0.08 | 1.34 | 0.32 | – | – | – | – |
|  | 7 | – | – | – | – | – | – | – | – | – | 0.00 | 0.23 | 0.23 | 0.39 | 0.12 | – | – | – | – |
| Ogur *et al*.^14^ | 3 | – | – | – | – | – | – | – | – | – | 0.00 | 0.10 | – | 0.30 | 0.00 | 0.40 | 0.20 |  | 2.90 |
| Mahjoub *et al*.^10^ | 1 | – | – | – | – | – | – | – | – | – | 2.17 | 10.07 | – | – | 0.20 | 1.18 | 8.05 | – | – |
|  | 2 | – | – | – | – | – | – | – | – | – | 3.20 | 7.57 | – | – | 0.64 | 2.56 | 5.40 | – | – |
|  | 3 | – | – | – | – | – | – | – | – | – | 2.24 | 5.50 | – | – | 1.09 | 1.18 | 6.10 | – | – |
|  | 4 | – | – | – | – | – | – | – | – | – | 1.54 | 8.90 | – | – | 1.54 | 2.57 | 7.90 | – | – |
|  | 5 | – | – | – | – | – | – | – | – | – | 2.06 | 6.66 | – | – | 0.26 | 1.07 | 4.20 | – | – |
| Mean (published data) | | 1.39 | 0.18 | 0.41 | 0.08 | 0.18 | 0.16 | 0.35 | 1.37 | 0.44 | 0.43 | 1.19 | 0.34 | 0.71 | 0.60 | 0.73 | 2.09 | 0.32 | 1.12 |
| SD (published data) | | 0.79 | 0.02 | 0.20 | 0.09 | 0.31 | 0.08 | 0.15 | 0.50 | 0.04 | 0.71 | 2.49 | 0.76 | 0.79 | 0.96 | 0.75 | 2.98 | 0.48 | 0.85 |
| Mean (present study) | | 0.39 | 0.44 | 0.34 | 0.18 | 0.17 | 0.39 | 0.29 | 0.35 | 0.21 | 0.18 | 1.91 | 1.13 | 0.63 | 0.59 | 0.61 | 1.11 | 0.59 | 0.92 |
| SD (present study) | | 0.26 | 0.49 | 0.26 | 0.16 | 0.14 | 0.27 | 0.33 | 0.26 | 0.07 | 0.18 | 1.59 | 0.63 | 0.18 | 0.34 | 0.60 | 0.51 | 0.36 | 0.68 |

SD, standard deviation.
