# Supplementary material for: FISH analysis of numerical chromosomal abnormalities in the sperm of robertsonian translocation der(13; 14)(q10;q10) carriers
Source: Front Genet. 2022 Sep 27;13:1010568. doi: 10.3389/fgene.2022.1010568 (PMC9551382; doi:10.3389/fgene.2022.1010568)
Supplement: Supplementary file 2 [file Table2.DOC]

| **Table 1 |** The analysis of segregation modes and numerical abnormality for translocated chromosomes 13 and 14. | | | | | | | | | | | | | | |
| --- | --- | --- | --- | --- | --- | --- | --- | --- | --- | --- | --- | --- | --- | --- |
| **Patient No.** | **Segregation modes** | | | | |  | **Numerical abnormality** | | | | | | | |
|  | **Alternate(%)** | **Adjacent (%)** | **3:00 (%)** | **Diploidy(%)** | **Others(%)** |  | **Nullisomy 13 (%)** | **Disomy 13 (%)** | **Aneuploidy 13 (%)** | **Total numerical abnormality for chromosome 13 (%)** | **Nullisomy 14 (%)** | **Disomy 14 (%)** | **Aneuploidy 14 (%)** | **Total numerical abnormality for chromosome 14 (%)** |
| 11 | 62.39 | 35.02 | 1.34 | 0.67 | 0.57 |  | 22.01 | 3.54 | 25.55 | 26.79 | 8.42 | 3.73 | 12.15 | 13.40 |
| 12 | 62.03 | 34.19 | 3.08 | 0.50 | 0.20 |  | 17.30 | 6.16 | 23.46 | 24.16 | 12.82 | 4.08 | 16.90 | 17.59 |
| 13 | 75.73 | 22.59 | 1.31 | 0.37 | 0.00 |  | 10.78 | 4.12 | 14.90 | 15.28 | 4.97 | 5.34 | 10.31 | 10.68 |
| 14 | 78.04 | 18.69 | 2.27 | 0.49 | 0.49 |  | 6.92 | 3.26 | 10.19 | 11.18 | 5.84 | 7.22 | 13.06 | 14.05 |
| 15 | 79.28 | 18.30 | 2.06 | 0.00 | 0.36 |  | 7.17 | 6.01 | 13.18 | 13.54 | 5.11 | 4.13 | 9.24 | 9.60 |
| 16 | 71.29 | 26.43 | 1.81 | 0.19 | 0.29 |  | 6.37 | 6.84 | 13.21 | 13.69 | 10.65 | 6.18 | 16.83 | 17.30 |
| 17 | 85.53 | 13.38 | 0.69 | 0.40 | 0.00 |  | 3.27 | 3.07 | 6.34 | 6.74 | 6.24 | 2.18 | 8.42 | 8.82 |
| 18 | 65.89 | 32.95 | 0.87 | 0.10 | 0.19 |  | 6.57 | 6.96 | 13.53 | 13.82 | 14.11 | 7.05 | 21.16 | 21.45 |
| 19 | 80.24 | 16.22 | 2.80 | 0.37 | 0.37 |  | 4.75 | 6.06 | 10.81 | 11.56 | 7.27 | 3.73 | 11.00 | 11.74 |
| 20 | 83.32 | 16.48 | 0.10 | 0.00 | 0.10 |  | 3.50 | 5.19 | 8.69 | 8.79 | 4.70 | 3.30 | 7.99 | 8.09 |
| Mean | 74.37 | 23.42 | 1.63 | 0.31 | 0.26 |  | 8.86 | 5.12 | 13.99 | 14.55 | 8.01 | 4.69 | 12.71 | 13.27 |
| SD | 8.10 | 11.63 | 0.90 | 0.22 | 0.19 |  | 5.85 | 1.42 | 5.80 | 6.00 | 3.23 | 1.59 | 4.09 | 4.14 |

| **Table 2 |** The frequency of numerical abnormalities for nontranslocated chromosomes and the results of comparison with donor controls. | | | | | | | | | | | | |
| --- | --- | --- | --- | --- | --- | --- | --- | --- | --- | --- | --- | --- |
| **Type of abnormality** | **Chromosome** | | | | | | | | | | | |
|  | 1 | 2 | 3 | 4 | 5 | 6 | 7 | 8 | 9 | 10 | 11 | 12 |
| Nullisomy | 0.57 | 0.6 | 1.64*** | 0.62*** | 0.3 | 0.98*** | 0.35 | 0.46 | 0.54** | 0.41 | 0.56** | 0.51*** |
| Disomy | 0.39*** | 0.44*** | 0.34*** | 0.29 | 1.08*** | 0.28** | 0.18** | 0.17 | 0.39*** | 0.17 | 0.23** | 0.29** |
| Diploidy | 0.69*** | 0.67* | 0.54*** | 0.48* | 0.45 | 0.48 | 0.61*** | 0.6*** | 0.52* | 0.57* | 0.57*** | 0.48 |
| Others | 0.01 | 0.03 | 0 | 0.05 | 0.00** | 0.03 | 0.04 | 0.01 | 0.04 | 0.03 | 0.01 | 0.04 |
| Aneuploidy | 0.96** | 1.04* | 1.97*** | 0.91*** | 1.38*** | 1.26*** | 0.54** | 0.63 | 0.93*** | 0.59 | 0.79*** | 0.79*** |
| Total numerical abnormality | 1.66*** | 1.74** | 2.51*** | 1.44*** | 1.83*** | 1.77*** | 1.19*** | 1.25*** | 1.48*** | 1.18* | 1.38*** | 1.31*** |
| **Type of abnormality** | **Chromosome** | | | | | | | | | | | |
|  | 15 | 16 | 17 | 18 | 19 | 20 | 21 | 22 | SEX | Mean | | SD |
| Nullisomy | 0.42 | 0.62*** | 0.2 | 0.61 | 0.42* | 1.24*** | 1.11*** | 0.59*** | 0.92*** | 0.65*** | | 0.34 |
| Disomy | 0.35*** | 0.16 | 0.21 | 0.18 | 1.39*** | 0.32 | 1.91*** | 1.13*** | 0.63** | 0.5*** | | 0.46 |
| Diploidy | 0.61 | 0.75* | 0.73** | 0.53** | 0.6 | 0.62*** | 0.65* | 0.6 | 0.75*** | 0.59*** | | 0.09 |
| Others | 0.01 | 0.01 | 0.03 | 0.03 | 0.03 | 0.02 | 0.06 | 0.04 | 0.07 | 0.03 | | 0.02 |
| Aneuploidy | 0.77*** | 0.78*** | 0.41 | 0.79 | 1.8*** | 1.56*** | 3.02*** | 1.73*** | 1.55*** | 1.15*** | | 0.6 |
| Total numerical abnormality | 1.39*** | 1.54*** | 1.16 | 1.35** | 2.43*** | 2.19*** | 3.73*** | 2.37*** | 2.36*** | 1.77*** | | 0.62 |
| ***p* < 0.05; ***p* < 0.01; ****p* < 0.001 | | | | | | | | | | | | |

| **Table 3 |** The frequency of total nullisomy, disomy, diploidy, others, and aneuploidy, and numerical abnormality per carrier with all chromosomes and with non-translocated chromosomes. | | | | | | | |
| --- | --- | --- | --- | --- | --- | --- | --- |
| **Type of abnormality** | **with all chromosomes (1**–**22, X and Y)** | | |  | **with nontranslocated chromosomes (1**–**12,15**–**22, X and Y)** | | |
|  | **der(13;14) carrier** | **control donor** | **p-value** |  | **der(13;14) carrier** | **control donor** | **p-value** |
| Nullisomy | 30.57±14.40 | 7.62±3.23 | 0.001 |  | 13.69±8.40 | 7.08±3.03 | 0.039 |
| Disomy | 20.35±5.95 | 3.63±1.18 | 0.000 |  | 10.54±4.08 | 3.21±0.91 | 0.000 |
| Diploidy | 0.57±0.22 | 0.36±0.15 | 0.025 |  | 0.59±0.23 | 0.36±0.15 | 0.015 |
| Others | 0.05±0.02 | 0.02±0.01 | 0.006 |  | 0.03±0.02 | 0.02±0.01 | 0.227 |
| Aneuploidy | 50.92±15.40 | 11.25±3.21 | 0.000 |  | 24.22±8.68 | 10.29±2.96 | 0.000 |
| Total | 51.53±15.45 | 11.63±3.19 | 0.000 |  | 24.84±8.65 | 10.67±2.93 | 0.000 |
